# Combating Reactive Oxygen Species (ROS) with Antioxidant Supramolecular Polymers

**DOI:** 10.1021/acsami.5c06967

**Published:** 2025-06-04

**Authors:** Penelope E. Jankoski, Zacchaeus M. Wallace, Loria R. DiMartino, Jessica Shrestha, Ashe M. Davis, Iyanuoluwani Owolabi, Alex S. Flynt, Tristan D. Clemons

**Affiliations:** † School of Polymer Science and Engineering, 5104 University of Southern Mississippi, Hattiesburg, Mississippi 39406, United States; ‡ Center for Molecular and Cellular Biosciences, 5104 University of Southern Mississippi, Hattiesburg, Mississippi 39406, United States; § School of Biological, Environmental, and Earth Sciences, University of Southern Mississippi, Hattiesburg, Mississippi 39406, United States; ∥ Department of Biomedical Engineering, University of Mississippi, Oxford, Mississippi 38677, United States

**Keywords:** reactive oxygen species, oxidative stress, peptide amphiphiles, antioxidant, tissue regeneration

## Abstract

Reactive oxygen species (ROS) are highly damaging biological molecules significantly upregulated following major injuries or diseases such as heart attack, burn injury, and stroke. Despite promising preclinical results, traditional small-molecule antioxidant therapies have had limited success in clinical applications. In this study, we employed a macromolecular approach to combat ROS, demonstrating that tethering the potent biological antioxidant, glutathione, to a peptide amphiphile effectively consumes harmful extracellular radicals while preserving antioxidant and polymeric functionality. By neutralizing these radical species, we can protect vulnerable cells from acute ROS toxicity. This was validated by assessing cellular oxidative damage and survival in cell lines stimulated with tert-butyl hydroperoxide (tBHP) to induce ROS production. The antioxidant nanofibers achieved cell rescue at concentrations an order of magnitude lower than molecular glutathione, a direct result of the extracellular localization and enhancement in the proximal concentration of the glutathione moieties along the supramolecular polymer. These antioxidant supramolecular polymers offer proof of principle for a macromolecular strategy to combat the damaging effects of extracellular ROS associated with disease and injury, showcasing their efficacy at low concentrations and maintaining antioxidant capabilities when in the gelled state, providing for the potential of an antioxidant tissue regenerative scaffold.

## Graphical abstract

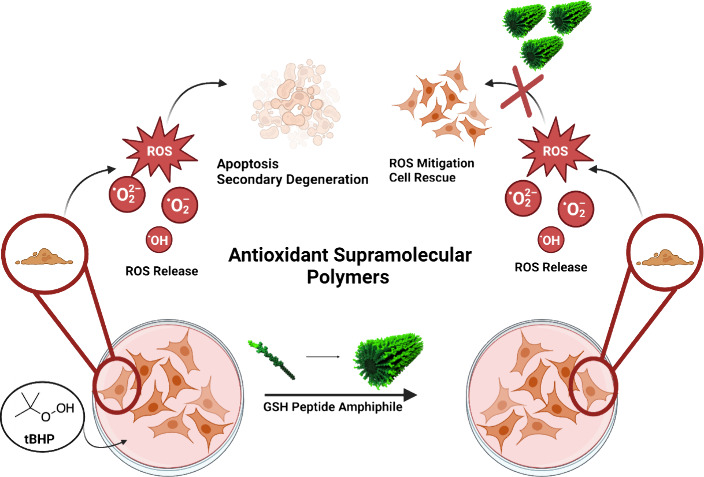

## 1 Introduction

The upregulation of reactive oxygen species (ROS)—including unstable compounds such as hydrogen peroxide, hydroxyl radicals, singlet oxygen, and superoxide—serve as a hallmark of many acute injuries and chronic diseases, resulting from increased cellular stress.^1,2^ While they play essential roles in cellular signaling and immune responses, their dysregulation can lead to oxidative stress, contributing to the pathogenesis of various diseases. Elevated levels of ROS have been implicated in a range of conditions, including cardiovascular disease, cancer, diabetes, burn wounds, and metabolic syndromes, affecting millions of individuals each year.^3,4^ For instance, chronic oxidative stress has been associated with the development and progression of atherosclerosis and hypertension.^5,6^ In a state of homeostasis, these molecules play a pivotal role in metabolic regulation, intercellular signaling, and the maintenance of antioxidant defenses. However, during pathological conditions, cells often become inundated with elevated levels of ROS, leading to oxidative stress, subsequent cellular damage, and cell death.^7,8^ In a process known as secondary degeneration, ROS released into the extracellular space further propagate injury to neighboring cells, perpetuating damage and contributing to large-scale tissue death and organ dysfunction.^9,10^ ROS, in excess, can overwhelm the body’s antioxidant defenses, leading to oxidative stress. This stress can cause damage to cellular components such as lipids, proteins, and DNA, initiating a cascade of inflammatory responses. For example, in cardiovascular diseases, oxidative stress contributes to endothelial dysfunction, promotes atherosclerotic plaque formation, and enhances vascular inflammation, leading to further tissue damage.^1,6^ Additionally, lipid peroxidation generates reactive byproducts that further propagate oxidative stress and inflammation, amplifying cellular damage. The release of these damaging radicals then activates inflammatory pathways, which can exacerbate tissue damage and further increase ROS upregulation.^11^ Thus, in these conditions, large-scale tissue regeneration is often required to support healing following ROS exposure. Animal models of acute cardiac ischemia/reperfusion (I/R) injury have shown that up to 50% of the final area of the damaged tissue directly arises from ROS associated with the reperfusion phase, which highlights both significant preventable damage, and the potential benefit of effective antioxidant therapies.^7^ Hardy et al., demonstrated in an *ex vivo* I/R injury model that locally treating with polymer nanoparticles loaded with a potent antioxidant resulted in a significant reduction in lactate dehydrogenase (LDH) release and creatine kinase (CK) levels,^12^ demonstrating the potential benefit of antioxidants in these types of injuries.

Current antioxidant treatments aim to use high concentrations of antioxidant molecules, mitochondrial scavengers, and peptide-based therapies to mitigate this oxidative burst. However, these have struggled to achieve clinical translation due to an inability to localize at the site of ROS upregulation at therapeutically relevant concentrations.^13–15^ After much enthusiasm in the 1980s and 1990s surrounding antioxidant therapies, few have successfully translated past clinical trials for the treatment and prevention of diseases.^12,16–19^ These materials, including small molecule antioxidants, nanoparticles, and enzymatic scavengers, all of which have demonstrated efficacy in reducing oxidative stress and preventing ROS-induced cellular damage. For example, nanomaterials such as cerium oxide nanoparticles and fullerene derivatives have been explored for their ability to scavenge ROS and protect tissues from oxidative damage.^20,21^ Similarly, other polymeric approaches to antioxidant materials have leveraged polymers with inherent antioxidant functionality including polymers such as poly­(dopamine),^22,23^ and poly­(phenols).^24^ Gianneschi, Lu and co-workers recently pioneered a bioinspired oxidative polymerization of allomelanin,^25^ creating highly antioxidant nanoparticles which were successful in demonstrating wound healing benefits in topical application for the treatment of radiation induced dermatitis.^26^ However, a major limitation of these approaches is their inability to provide scaffolding to enhance antioxidant localization and to promote tissue regeneration. Specifically, these antioxidants typically lack the necessary properties to support cell growth, differentiation, and tissue remodeling—coupled with the antioxidant potential. Recent advancements in the development of antioxidant-loaded biomaterials, such as hydrogels and biodegradable polymers, are addressing this gap by incorporating antioxidants into scaffolds that promote cellular regeneration while mitigating ROS.^27,28^

Other work has sought to harness extracellular ROS as a stimuli in the development of stimuli-responsive materials.^29,30^ Recent advances have focused on utilizing inherently antioxidant amino acids, such as methionine, to protect cells from oxidative damage. Notably, Muraoka et al. demonstrated the potential of a methionine-rich peptide that, over several days, could self-assemble and gel, providing effective protection to Jurkat cancer cells from H_2_O_2_-induced ROS cell death. The oxidation of these materials triggered a transition from gel to solution, highlighting their dynamic response to oxidative stress.^31^ Similarly, ROS have been used successfully in the targeted release of therapeutics at an injury site and for the breakdown of tissue regeneration scaffolds, primarily utilizing degradable peptide and polymer chemistries.^27,32,33^ Additionally, other studies have explored leveraging ROS to trigger the release of bioactive molecules from scaffolds, thereby creating a degradable system that not only supports tissue regeneration but also facilitates wound healing by delivering anti-inflammatory agents.^34^ While advancements in leveraging reactive oxygen species (ROS) for therapeutic delivery and tissue regeneration have shown promise, there remains a critical need for materials that can both mitigate ROS-induced damage and maintain their physical properties to provide structural support during the healing process. This underscores the importance of developing strategies that combine antioxidant functionality with robust structural frameworks to more comprehensively address oxidative stress.

Peptide amphiphiles (PAs) are an exciting class of biomaterial for tissue regeneration applications due to their biocompatibility, biodegradability, and ability to mimic the scaffolding structure of the extra cellular matrix with their filamentous supramolecular assembly.^35,36^ Peptide amphiphiles, borrowing tricks from proteins such as hydrophobic interactions, β-sheet hydrogen bonding, and charge repulsion are programmed to self-assemble into elongated nanofibers. These nanofibers are shear thinning and able to rapidly self-heal following breakage allowing for, in the presence of ionic cross-linking, the ability for injection delivery or 3D-printing of self-supporting architectures.^37,38^ Depending on concentration and processing, these materials have been used in a number of biomaterial applications ranging from targeted therapies,^39–42^ through to cellular scaffolds for tissue regeneration applications.^38,43–45^ Motivated by the lack of clinically approved therapies for the effects of ROS in disease states, we aimed to utilize this highly modular platform to design an antioxidant supramolecular polymer that utilizes the glutathione tripeptide to provide antioxidant therapy with the highly tunable PA nanofiber platform as a ROS-capturing biomaterial.

## 2 Materials and Methods

### 2.1 Materials

Acetonitrile, Arsenazo III, dichloromethane (DCM), *N*,*N*-diisopropylethylamine (DIEA), *N*,*N*-dimethylformamide (DMF), diethyl ether, methanol, 4-methylpiperidine, sodium hydroxide (NaOH) were all purchased from Sigma-Aldrich and used as received. Palmitic acid was obtained from Acros Organics, and used as received. All Fmoc-protected amino acids, Oxyma, and Rink amide MBHA resin were purchased from CEM. Triisopropylsilane (TIPS), trifluoroacetic acid (TFA), phosphate buffered saline (PBS), Dulbecco’s modified eagle medium (DMEM), heat inactivated fetal bovine serum (FBS), penicillin-streptomycin, he CyQUANT Lactate Dehydrogenase (LDH) Assay, Live/Dead Imaging kits, and CellRox Deep Red fluorescent probe were all purchased from ThermoFisher. Sterile tissue treated 6 and 96-well plates were obtained from CellTreat USA. Nile Red was purchased from ApexBio and 2,2-Diphenyl-1-picrylhydrazyl (DPPH) was obtained from the Cayman Chemical Company.

### 2.2 Peptide Amphiphile Synthesis and Preparation

#### 2.2.1 Peptide Amphiphile Synthesis

Peptide amphiphiles (PAs) were synthesized using standard Fmoc-solid-phase peptide chemistry as we have described previously.^46,47^ PAs were synthesized on Rink amide MBHA resin with the amino acid couplings performed on a CEM Liberty Blue microwave-assisted peptide synthesizer (CEM, Matthews, NC, USA). Fmoc groups were cleaved using 20% 4-methylpiperidine in *N*,*N*-dimethylformamide (DMF) at 90 °C for 30 s. Amino acids were coupled using 4 mol equivalent (equiv) of each Fmoc-protected amino acid, 8 equiv ethyl cyanohydroxyiminoacetate (Oxyma) and 8 equiv of *N*,*N*′-diisopropylcarbodiimide (DIC) for 2–4 min at 90 °C in DMF as solvent. Using this same procedure, palmitic acid (C_16_) was conjugated to the N-terminus of the peptide as the hydrophobic tail. Completed PA molecules were cleaved off the resin using a solution of 95:2.5:2.5 trifluoroacetic acid (TFA)/triisopropylsilane (TIPS)/water for 2–3 h at ambient temperature on a wrist shaker. Following cleavage, PAs were precipitated with cold diethyl ether, collected via centrifugation, and dried overnight. The PAs were then purified by preparative scale reverse phase high performance liquid chromatography (CEM Prodigy HPLC) using a Phenomenex Gemini column (C-18 stationary phase, 5 μm, 100 Å pore size, 50 × 250 mm). A mobile phase of acetonitrile and water was used, both containing 0.1% NH_4_OH. Pure fractions were identified using electrospray ionization mass spectroscopy (ESI-MS) in negative ion mode on a ThermoScientific Orbitrap ExplorisTM 240 mass spectrometer using direct injection. Excess acetonitrile was removed with rotary evaporation, freeze-dried, and the powders stored at −20 °C until use.

#### 2.2.2 Peptide Amphiphile Preparation

All PAs were obtained from their lyophilized powder form and reconstituted in deionized (DI) water to achieve a final concentration of 10 mM. The pH was adjusted to 7.4 with 1 M NaOH. The samples were then lyophilized again and stored at −20 °C until use. PAs were diluted from these stock solutions and utilized “as is” unless otherwise specified. For gelation, a solution comprising 125 mM NaCl, 3 mM KCl, and 25 mM CaCl_2_ in DI water was employed. PAs were initially plated, and an equal volume of the gelling solution was added. After a 15 min incubation period, the excess gel solution was removed, resulting in an ionically gelled network.

### 2.3 Material Characterization

#### 2.3.1 Liquid Chromatography Mass Spectrometry (LC-MS)

The purity of PA molecules was confirmed using liquid chromatograph-mass spectroscopy (LC-MS), which was performed using an Agilent 1200 system with a Phenomenex Gemini C-18 column (100 × 1.00 mm; 5 μm) for basic conditions. The mass detector (MS) was an Agilent 6520 Q-TOF MS. All gradient methods followed: acetonitrile at 5% for 5 min at 50 μL/min, 5–95% over 25 min at 50 μL/min followed by 95% for 5 min at 50 μL/min. Ammonium hydroxide (0.1% v/v) for basic conditions was added to all solvents. Peaks were detected at λ = 220 nm.

#### 2.3.2 Nuclear Magnetic Resonance (NMR) Spectroscopy

Structural analysis of the GSH PA and Control PA through ^1^H and ^13^C NMR was performed using a Bruker 600 MHz spectrometer in deuterated TFA (*d*1) at ∼50 mg/mL. Chemical shifts are in ppm relevant to the solvent peak.

#### 2.3.3 Nile Red Assay

Nile Red stock solutions were prepared at 10 mM in DMSO and subsequently diluted inDI water to achieve a final working concentration of 100 μM. PA samples were diluted in DI water to generate a concentration range of 0–500 μM. In a 96 well plate at each concentration, 90 μL of PA solution and 10 μL of Nile Red solution were added to each well, mixed thoroughly and allowed to incubate at room temperature for 3 h, with intermittent tapping to enhance incorporation. Each sample was performed in triplicate and following incubation, the samples were analyzed using a Biotek Synergy H1Microplate Reader (Agilent), with excitation set at 550 nm and emission monitored in 2 nm increments from 580 to 720 nm. The maximum relative fluorescence units (RFU) were plotted against the logarithm of the concentration, and the critical aggregation concentration (CAC) was determined as the intersection point of the curves representing no fluorescent signal and the presence of the fluorescent signal.

#### 2.3.4 Transmission Electron Microscopy (TEM)

Samples were prepared on 300-mesh copper grids with a continuous carbon film (Electron Microscopy Sciences, Hatfield, PA, USA). Peptide amphiphile samples were prepared at a concentration of 0.5 mM, and 3 μL of the solution was drop-cast onto the grid and allowed to air-dry. After drying, the samples were incubated with 1% uranyl acetate for 30 s, followed by the removal of excess solution by wicking with filter paper to enhance image contrast. Transmission electron microscopy (TEM) was conducted on a JEOL JEM120i instrument at 120 kV.

#### 2.3.5 Circular Dichroism (CD)

Samples were prepared as previously described (Section 2.2.2). Each sample was diluted to concentrations of 500, 250, and 125 μM in DI water. CD spectra were recorded on a JASCO model J-815 spectropolarimeter using a quartz cell of 0.5 mm optical path length. Continuous scanning mode was used with a scanning speed of 100 nm per minute with the sensitivity set to standard mode. High Tension (HT) voltage was recorded for each sample to ensure that the measurement was not saturated. An accumulation of three measurements was used and a buffer sample was background-subtracted to obtain final spectra. The final spectra were normalized to the concentration of each sample.

#### 2.3.6 Rheological Measurements

Rheological measurements were conducted using a strain-controlled ARES rheometer (TA Instruments) fitted with 25 mm cone and plate steel geometry, maintaining a gap height of 0.3 ± 0.5 mm. To establish the viscosity profile of the material, a steady rate sweep test was performed across a shear rate range of 0.001–10 s^–1^. Uncross-linked samples at a concentration of 10 mM were prepared as described in Section 2.2.2. To assess the effects of ionic cross-linking on the viscosity of the PA samples, 450 μL of fresh PA was placed on the steel plate, followed by an equal volume of gelling solution pipetted on top. The samples were allowed to gel for 15 min, after which 450 μL of gelling solution was carefully removed, and the test was performed as above. Measurements about the storage and loss modulus, as well as the thixotropic behavior of the samples were made using the dynamic motor mode, with cross-linked 10 mM PA samples as described above. The PAs were investigated using a dynamic strain sweep from 0.1–50% Strain. Then a frequency sweep from 1–100 rad/s was performed at a fixed strain of 1%. Finally, thixotropic behavior was investigated using a seven-step oscillatory method, where frequency and strain were fixed, and samples were held for 50 s while monitoring *G*′/*G*″ every 10 s in a time sweep. To determine the impact of oxidation on material properties, samples were oxidized using 2% H_2_O_2_ with a 15 min incubation in air at room temperature. 2 mM samples were then run on the rheometer with a steady rate sweep test performed across a shear rate range of 0.01–10 s^–1^ and samples were recovered from the rheometer and used in the DPPH assay as described below.

#### 2.3.7 Scanning Electron Microscopy (SEM)

SEM stubs were prepared with conductive carbon tape and 250 μL of PA sample was added. They were then submerged in liquid N_2_ until completely frozen (∼30 s) and lyophilized. Samples were then analyzed using a Sigma VP Field Emission scanning electron microscope (Zeiss). For gelled samples, gelation solution was applied, and samples were gelled on carbon tape following protocol as described in Section 2.2.2 and left to incubate for 15 min. Excess gelling solution was then removed prior to freezing and lyophilization.

#### 2.3.8 Ca^2+^ Release Assay

GSH PA hydrogels (10 mM, 200ul) were prepared in 1.5 mL Eppendorf tubes as described in Section 2.2.2 and allowed to ionically cross-link. Following incubation with the gelling solution, the solution was removed, and the hydrogels were washed twice with 1 mL of DI water, before the addition of 400 μL of phosphate buffered saline (PBS, without divalent cations) onto the hydrogels as the sink solution. The samples were incubated at 37 °C with 10 μL aliquots collected from the sink at defined time points (0–24 h). Aliquots were frozen at −20 °C until completion of the kinetics, thawed and then Ca^2+^ concentration quantified with the Arsenazo III assay. At mildly acidic pH, metallo-chromogen Arsenazo III combines with calcium to form a colored complex which absorbs intensely at 650 nm proportionally to the amount of Ca^2+^ in the sample. The Arsenazo III reagent (500 μL of 0.2 mM Arsenanzo III prepared in a 0.1 M, pH 6.8 Imidazole buffer) was directly added to the 10 μL of sample aliquots, mixed vigorously with a vortex mixer for 10 s before adding to a 96 well plate to measure the absorbance at 650 nm. Final Ca^2+^ concentration was determined through comparison to a standard curve made with known standards of CaCl_2(aq)_ prepared from 0 to 10 mM.

#### 2.3.9 2,2-Diphenyl-1-picrylhydrazyl (DPPH) Assay

DPPH was prepared in methanol at a concentration of 50 μM and stored protected from light at −20 °C. PA samples were prepared as outlined in Section 2.2.2 and diluted to the desired working concentrations using DI water. For each concentration, samples were performed in triplicate and mixed with 50 μM DPPH in methanol at equal volumes in a 96-well plate. The samples were incubated in the dark at room temperature for 30 min before being analyzed on a microplate reader (Biotek Synergy H1) at 517 nm. To normalize the data, two controls were included: three wells containing a water–methanol mixture to account for solvent effects on absorbance, and three wells containing DPPH in water, serving as the 100% DPPH radical activity control.

### 2.4 *In Vitro* Experiments

#### 2.4.1 Cell Culture

Human embryonic kidney cells (HEK 293) were cultured in Dulbecco’s Modified Eagles Medium (DMEM) which was supplemented with 10% fetal bovine serum (FBS) and 1% Penicillin-Streptomycin. Cells were cultured at 37 °C and 5% CO_2_ and used when confluence in the flask reached 80%.

#### 2.4.2 *In Vitro* ROS Generation Assay

Cells were harvested from culture flasks using a Trypsin-EDTA solution, which was subsequently diluted with enriched DMEM, followed by centrifugation. The resulting cell pellet was resuspended in enriched DMEM and diluted to a concentration of 1 × 10^5^ cells/mL, confirmed using trypan blue staining and a Countess Cell Counter (Invitrogen). To assess the optimal concentration of tert-butyl hydroperoxide (tBHP) for inducing cell death and extracellular ROS production, cells were plated in a 96-well plate at a volume of 100 μL per well and allowed to adhere overnight. A stock solution of tBHP was prepared by diluting aqueous tBHP in nuclease-free water. Various volumes of the tBHP stock solution were then added to the wells to achieve final concentrations ranging from 0 to 100 μM, with a minimum replicate of *n* = 9 for each concentration. The cells were incubated at 37 °C in a 5% CO_2_ atmosphere for 24 h. A spontaneous control was included, wherein cell culture media was replaced with nuclease-free water only. Following the 24 h exposure, a Lactate Dehydrogenase (LDH) assay was conducted according to the manufacturer’s instructions (Invitrogen CyQUANT kit). To determine the propensity of the GSH PA and the Control PA to rescue cells from extracellular radicals, they were added at the same time as tBHP and coincubated for 24 h.

#### 2.4.3 Live Cell Imaging

To assess cell viability, Live/Dead Imaging was performed using a Live/Dead Cell Imaging kit (ThermoFisher) following the kit protocol. Briefly, cells were cultured in a glass bottom, black-welled, 96-well plate, and the ROS Assay was performed *vide supra*. After 24 h, 70 μL of supernatant was removed from each well and 30 μL of Live/Dead stain was added. Cells were incubated at room temperature for 15 min prior to imaging on a STELLARIS STED Super-Resolution Confocal Microscope (Leica). In tBHP only wells, to confirm that the induced cell death was a product of ROS upregulation, the CellRox Deep Red Oxidative Stress stain was used. Cells were cultured in a glass bottom, black-welled, 96-well plate, and the ROS Assay was performed *vide supra*. Following 24 h tBHP exposure, CellRox stock was diluted with DMSO to 0.1 mM and 5 μL was added to each well to be at a final concentration of 5 μM. The cells were incubated at 37 °C for 30 min and then visualized on a STELLARIS STED Super-Resolution Confocal Microscope (Leica) with brightfield overlay to visualize the cells with the oxidative stress marker.

#### 2.4.4 Fixed Cell Imaging

Coverslips were prepared by rinsing for 2 h on the benchtop in a solution of ethanol, water, and sodium hydroxide (v/v% 57:33:10). Following this treatment, coverslips were washed three times with DI water and sterilized under two cycles of UV light (10 min per cycle). Coverslips were then placed in a 6-well plate and exposed to UV light for an additional two cycles. Coverslips were coated with a 0.1 mg/mL solution of poly-d-lysine and incubated at 37 °C for 24 h. Excess polylysine was removed, and the wells were rinsed with sterile PBS three times. HEK293 cells were plated at a density of 1.5 × 10^5^ cells/mL, 1.5 mL per well and allowed to adhere overnight. Treatments (tBHP and PAs) were added to the wells, maintaining a constant volume of 1.5 mL, and left to incubate for 24 h. Subsequently, CellRox Oxidative Stress Stain was applied for 30 min, after which the supernatant was removed, and the cells were washed three times with PBS. Cells were then fixed with 4% formaldehyde for 20 min. Following fixation, formaldehyde was removed, and the slides were rinsed three times with PBS. The coverslips were incubated with phalloidin iFluor for 45 min, rinsed three times, and mounted onto glass slides using DAPI mounting medium. Slides were imaged using a STELLARIS STED Super-Resolution Confocal Microscope (Leica) at 63x, ensuring consistent gain and intensity settings for the CellRox channel at 14 and 5.75%, respectively, to accurately compare the oxidative stress signals between samples. Quantification of oxidative stress was performed through image analysis of images collected at 20× magnification, nine total images for each sample. CellRox positive pixels were quantified using image analysis software (FiJi) as described previously.^48^ Both the count of positive pixels and the percent area covered by positive pixels were quantified, and normalized to the number of cell nuclei in the image.

## 3 Results and Discussion

### 3.1 Design of Glutathione (GSH) Functionalized Supramolecular Polymers

To achieve supramolecular polymers with antioxidant capabilities, we first designed a PA monomer suitable for supramolecular polymerization that would mimic the potent antioxidant capabilities of glutathione. This PA consisted of palmitic acid conjugated to three valine and three alanine residues to promote molecular aggregation and intermolecular β-sheet hydrogen bonding, respectively, and finally, three glutamic acid residues to impart aqueous solubility and amphiphilicity to the molecules. Coupled with this terminal glutamic acid is the tripeptide sequence Glu-Cys-Gly with a gamma peptide linkage between the carboxylic acid of the glutamic acid side chain and the cysteine residue to accurately mimic glutathione (GSH PA, Figure 1A). The GSH PA is capable of antioxidant quenching through the same pathway as molecular glutathione (GSH), where an electron from glutathione is donated to neutralize free radicals. Two oxidized glutathione molecules form the GSSG dimer through a disulfide bond.^49,50^ The PA maintains this mode of action through the glutathione mimetic moiety presented at the surface of the nanofibers, and it is anticipated that the oxidized structure could form between adjacent nanofibers (i.e., interfiber) or adjacent PA monomers within the same nanofiber (i.e., intrafiber). An identical PA monomer, lacking antioxidant functionality, was also synthesized as a control molecule (Control PA, Figure 1B). Liquid chromatography-mass spectrometry (LC-MS) indicated a high degree of purity for the target molecules with only a small percentage of oxidation of the GSH PA observed during the solid-phase peptide synthesis and purification process (see Figures S1 and S2 for LC-MS characterization). Furthermore, carbon and proton nuclear magnetic resonance (NMR) spectroscopy of the Control PA and GSH PA supports the successful synthesis of the PA monomers, with characteristic shifts in the carbon spectra supporting the inclusion of the GSH moiety (Figures S3 and S4).

**Figure 1:**
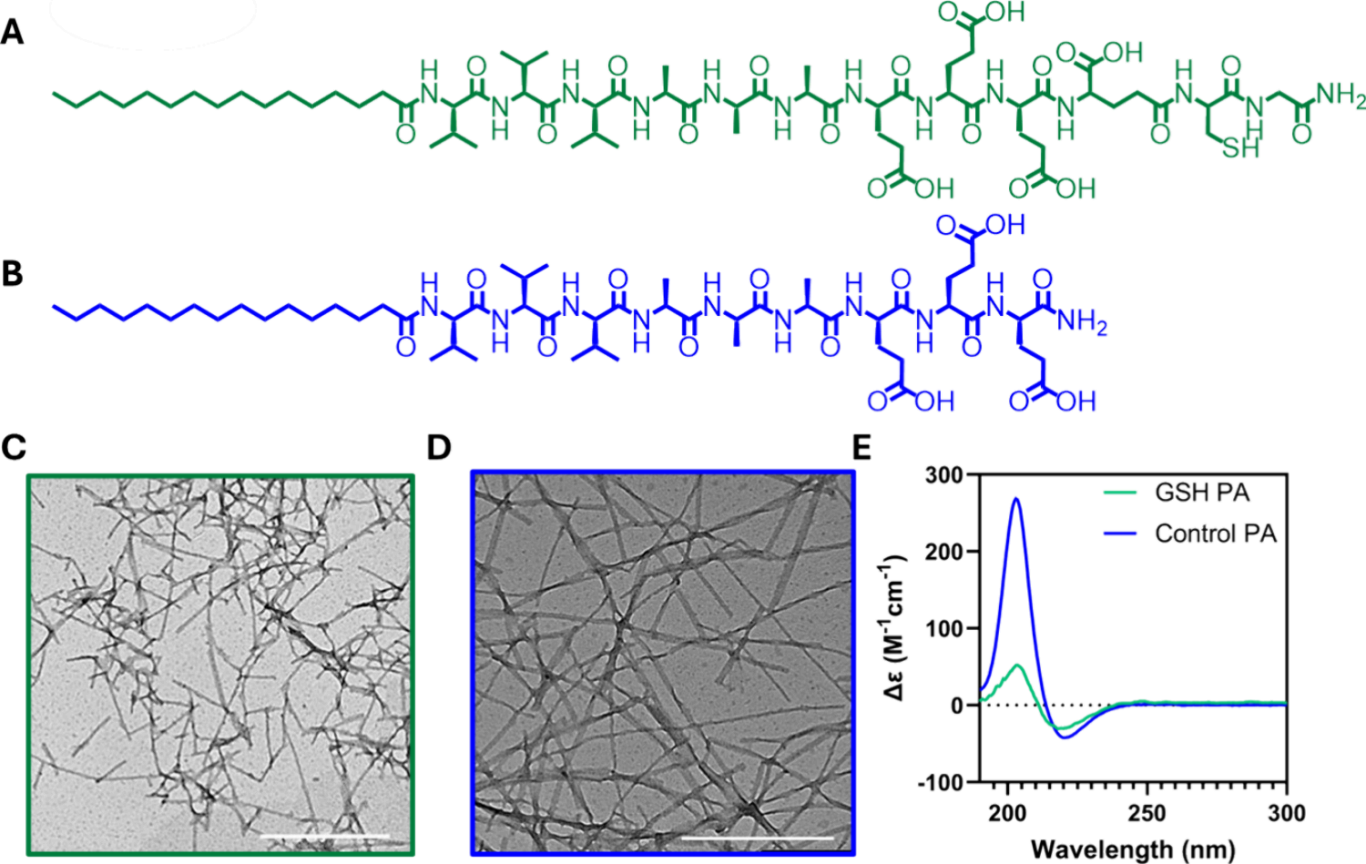
Supramolecular polymerization of the GSH and Control PA samples. Molecular structures of (A) the antioxidant GSH PA and (B) the Control PA without antioxidant potential. Transmission electron microscopy (all scale bars are 500 nm) of (C) GSH PA, and (D) Control PA. (E) Circular dichroism spectra of the GSH PA (green), and the Control PA (blue).

Supramolecular polymerization of the PA monomers into nanofibers was confirmed by transmission electron microscopy (TEM) where elongated nanofibers spanning microns in length for both the GSH PA (Figure 1C) and Control PA (Figure 1D) were observed, indicating that the incorporation of the glutathione tripeptide does not impede nanofiber formation. However, the GSH PA overall appeared to form shorter nanofibers when compared to the fiber-forming Control PA nanofibers. To investigate the internal arrangement and packing of molecules within the supramolecular polymers, circular dichroism (CD) spectroscopy was used to assess secondary structure. For both the GSH PA and Control PA supramolecular polymers, a characteristic β-sheet secondary structure was observed (Figure 1E), consistent with previous studies on similar molecules.^38,51,52^ Although the CD spectra for each molecule were similar, the GSH PA nanofibers exhibited a broader minimum and lower amplitude maxima compared to the Control PA. This observation, along with TEM observations, suggests that the GSH PA has a less pronounced long-range order relative to the Control PA. The Control PA displays a larger amplitude maximum, attributable to greater differences in ellipticity associated with longer fibers, and a sharper minimum due to reduced conformational freedom resulting from stronger β-sheet interactions.^51^ This finding is consistent with the observation of shorter fibers by TEM suggesting that the bioactive sequence of the GSH PA, with an additional carboxylic acid relative to the Control PA, presents with higher overall molecular charge and in turn greater molecular conformational freedom, that results in less efficient packing over extended length scales.^38,53^ Finally, a Nile red assay was used to probe the critical aggregation concentration (CAC) of both the GSH and Control PA nanofibers. The fluorescence from Nile Red, a solvatochromic fluorescent dye, is significantly enhanced in hydrophobic environments like those observed in the core of PA supramolecular polymers.^39,51^ The CAC for the GSH PA and the Control PA were calculated to be 5.1 and 6.6 μM respectively (Figure S5), demonstrating a high propensity for molecular self-assembly and organization within an aqueous environment. As anticipated the GSH tripeptide alone demonstrated no measurable CAC, even up to a concentration of 500 μM, suggesting no ability for the hydrophilic tripeptide to undergo self-assembly (Figure S5). Taken together, these results confirm that the presence of the GSH functionality does not appreciably impact the supramolecular polymerization of PA monomers into an elongated nanofiber. We hypothesize that this structure provides the potential for an antioxidant functionalized biomaterial that can be delivered in solution or via gel for the mitigation of biologically derived ROS.

### 3.2 Hydrogel Formation of (GSH) Functionalized Supramolecular Polymers

To assess the gelation properties of the PA nanofibers, we investigated the capability of the GSH PA and Control PA to form ionically cross-linked hydrogels that could be suitable for tissue regeneration applications. To achieve this, PA nanofibers were gelled with calcium ions, where the divalent cation produces ionic cross-linking between terminal glutamic acid residues of the PA nanofibers.^38^ By modulating the concentrations of PA nanofibers and calcium ions, one can effectively alter the resulting hydrogel’s viscosity and gelation, changing the stiffness and rheological profile of the biomaterial.^37^ A small amount of water-soluble food coloring was added to the nanofibers to enhance the imaging of the hydrogels. In the presence of calcium, the Control PA (blue) and GSH PA (green) both formed self-supporting hydrogels (Figure 2A). However, it appeared that the GSH PA formed a weaker gel due to the qualitative observation of a small amount of material not remaining in the gel following vial inversion. This is likely a result of the shorter nanofibers observed by TEM, and reduced supramolecular polymer entanglement, lowering the solution’s gelation capabilities. This is further supported by the inability to form gels at lower concentrations relative to the Control PA which was quantified using viscosity measurements from cone–plate rheology (Figure S6).

**Figure 2:**
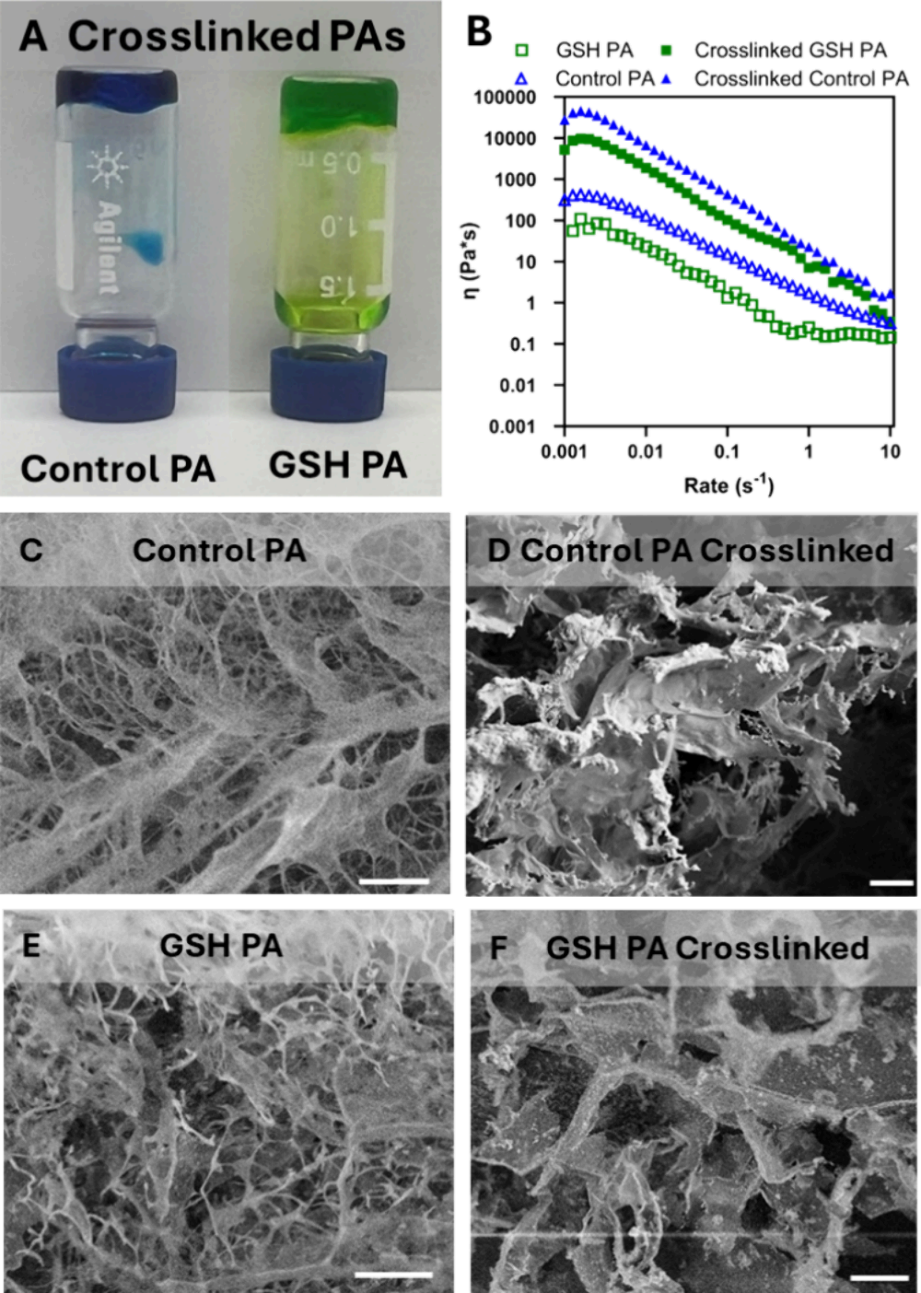
Characterization of ionic cross-linking of PA nanofibers and the resulting physical properties. (A) Photographs of PA nanofiber solutions in inverted vials of the self-supporting hydrogel of the Control PA (blue) and GSH PA (green) following gelation with the addition of Ca^2+^. To visualize the gels formed, 2 μL of food coloring was added to prepared PA nanofibers before Ca^2+^ addition. (B) Shear rate sweep from 0.001 to 10 s^–1^ to display viscosity differences between samples. Scanning electron microscopy (SEM) of (C) Control PA at 10 mM, (D) calcium cross-linked Control PA at 10 mM, (E) GSH PA at 10 mM, and (F) calcium cross-linked GSH PA at 10 mM. All scale bars are 20 μm.

The viscosity differences of the PA nanofiber solutions were assessed using a steady rate sweep test at a fixed strain, focusing initially on the PA nanofibers without calcium addition and a PA nanofiber concentration of 10 mM (Figure 2B). At low shear rates, a distinct hump in viscosity was noted, indicating that the materials were primarily outside the shear-thinning regime, allowing for relaxation of the induced shear. All tested materials exhibited shear-thinning behavior, characteristic of supramolecular polymers, which can disentangle and disassemble under high shear conditions but subsequently reassemble after the shear event.^54^ Additionally, the ionic gelation present in the materials was effectively disrupted under these conditions. Notably, no plateau in viscosity was observed in the Control PA in the absence of ionic cross-linking across the shear rates tested, compared to the GSH PA which had a plateau onset which shifts following cross-linking. Subsequently, fresh 10 mM samples were ionically cross-linked on the plate with the addition of gelling solution, and the same rheological test was conducted. The results indicated a significant increase in viscosity for the Control PA because of successful ionic cross-linking. A plateau at high shear rates was observed, suggesting the material stabilizes and maintains shear-independent behavior. Although the GSH PA formed a hydrogel at 10 mM in the presence of calcium as seen in Figure 2A, the gelation resulted in a lower observed viscosity compared to that of the Control PA. Ionic cross-linking of the GSH PA shifts the plateau to higher shear rates, indicating that a higher strain rate is required to achieve shear-independent behavior in these more viscous, gelled samples.

The surface topography of the PA nanofibers was assessed by scanning electron microscopy (SEM), comparing the nanofibers both in the presence and absence of supplemental calcium. Without added calcium, both the Control and GSH PA nanofibers formed an interconnected, 3-dimensional fibrous scaffold (Figure 2C–F). The ionic cross-linking of these materials results in thicker bundled structures being observed as PA nanofibers are pulled together through the ionic chelation of adjacent glutamic acid residues across nanofibers (i.e., internanofiber). At lower PA concentrations, the images are sparser resulting from the pulling together of fibers into larger bands and less bundling of the GSH PA nanofibers was observed relative to the Control PA (Figure S7). This established the ability of both the bioactive GSH PA and Control PA to ionically gel and form scaffolds, with impacts on fiber packing, length, and resulting rheological properties dependent on processing conditions. Importantly, an interconnected fibrous network was formed by both PAs providing a basis for the use of these materials as extracellular scaffolds that have the potential to scavenge ROS and protect cells.

We next sought to explore the gel-like properties of these ionically cross-linked materials to assess their utility as antioxidant hydrogels for potential tissue regenerative scaffolds (Figure 3). To achieve this, we conducted a dynamic strain sweep at a fixed frequency of 6.28 rad/s, aiming to define the linear viscoelastic region (LVR). Notably, the LVR for the GSH PA hydrogel is significantly broader compared to the Control PA hydrogel (Figure 3A). This broader LVR indicates that the GSH PA hydrogel can maintain its structural integrity and exhibit stable gel-like behavior over a wider range of applied strains. However, it is also evident that the GSH PA hydrogel demonstrates relatively lower values for both the storage modulus (*G*′) and loss modulus (*G*″), suggesting a weaker hydrogel overall. In contrast, the Control PA hydrogel exhibits higher moduli but shows a much narrower LVR, with its behavior deviating from that of an elastic solid at relatively lower strains. This disparity underscores the ability of the GSH PA gel to withstand greater strain variations while maintaining a gel-like consistency, despite its lower stiffness.

**Figure 3:**
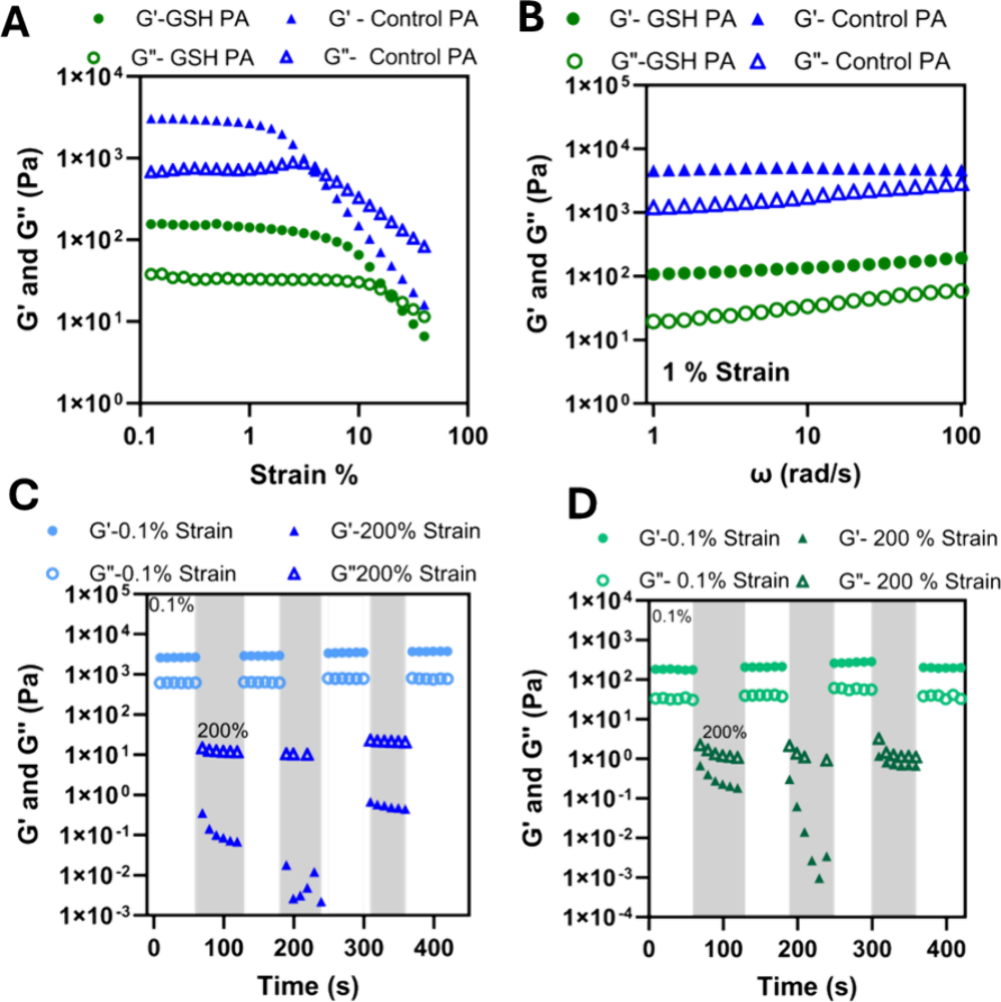
Characterization of rheological properties of the Control PA and GSH PA hydrogels. (A) Dynamic strain sweep of the Control PA (blue) and GSH PA (green) hydrogels from 0.1–50% strain to determine the linear viscoelastic region (LVR). (B) Frequency sweep at a strain of 1%, within the LVR, for the Control PA (blue) and GSH PA (green) hydrogels. Seven-step oscillation method to determine thixotropic behavior and ability to recover following high strain outside the LVR of the (C) Control PA, and (D) GSH PA hydrogels.

The storage and loss moduli of these hydrogels were then compared at a fixed strain within each hydrogel’s LVR, which was determined to be a strain of 1%. This region should have a *G*′ dominating the *G*″ and relative stability over frequencies. The Control PA had a *G*′ that was one hundred times greater than that of the GSH PA hydrogel (Figure 3B), which supported the visual observation of a stiffer hydrogel (Figure 2A), and the much higher observed viscosity (Figure 2B). We propose the longer more entangled, and tightly packed nanofibers, with the enhanced internal molecular ordering of the Control PA produce a stiffer hydrogel. In contrast the GSH PA nanofibers appear shorter by TEM, with reduced potential for entanglements, and reduced molecular ordering resulting in a lower storage modulus, even within the elastic regime (Figure 3B). To determine the propensity for these materials to recover following high strain, such as what would be sustained during injection or syringe delivery, a thixotropy test was performed using a seven-step oscillation method where stepwise deformations were applied within, and well above the LVR. For both PAs, 0.1% was selected as the low strain, and 200% was selected to mimic the high strain associated with injection from a syringe (Figure 3C,D). In the high-strain scenarios, *G*″ dominates as liquid-like behavior is observed and the material is completely deformed. The following low strain is then applied, and elastic modulus recovery is monitored providing an insight into the self-healing capability of the material. Highly thixotropic materials regain the elastic modulus that was seen prior to the application of high strain which indicates recovery following shear, important for injection delivery through a syringe. Both the Control PA and GSH PA experienced full recovery following high deformation strains, highlighting the self-healing nature of these biomaterials and the feasibility of needle or arthroscopic delivery of these hydrogels. The injectability of these materials was then experimentally validated through a subcutaneous injection into a chicken drumstick (Figure S8, Videos S1 and S2). Both the control and GSH PA were capable of injection through an 18G needle, with localized delivery around the injection site, maintaining location even with manipulation of the gel externally.

### 3.3 Antioxidant Capabilities of the GSH PA Nanofibers

The antioxidant capacity of the PA nanofibers to neutralize harmful ROS was evaluated using the 2,2-diphenyl-1-picrylhydrazyl (DPPH) radical scavenging assay. DPPH is a stable radical that exhibits strong absorption at 517 nm, presenting with a distinct purple color.^55^ Upon interaction with antioxidant compounds, the DPPH radical is quenched, resulting in a measurable color change, transitioning from purple to colorless. This colorimetric change allows for quantification of radical scavenging capability and serves as an effective indicator of a materials antioxidant activity. Since the color change of DPPH can be observed visually, we looked to investigate the ability of PA nanofibers to maintain antioxidant functionality as hydrogels (Figure 4A,B). The hydrogel itself impacts the absorbance and therefore cannot be read directly on a spectrophotometer, however, the visual change when the radical is fully quenched is a stark qualitative indicator. After 30 min, the Control PA hydrogel solution remained purple (Figure 4A) indicating no radical quenching, while the DPPH solution with the GSH PA hydrogel was visibly quenched to a clear, off-yellow color (Figure 4B). Importantly, this establishes that the GSH PA nanofibers maintain antioxidant functionality as a hydrogel following ionic cross-linking. At low PA concentrations without the presence of calcium ions and the formation of hydrogels, the antioxidant activity can be quantified with a spectrophotometer where the GSH PA demonstrated significant radical quenching relative to the Control PA nanofibers, remaining as impactful as untethered glutathione at all concentrations tested (Figure 4C). This further indicates that the antioxidant capabilities of GSH within the PA are not hindered after being tethered and localized within the PA nanofiber. The antioxidant activity of glutathione operates through a mechanism wherein two molecules of glutathione interact with a radical to form a dimer via a radical oxidation process, thereby neutralizing the radical.^49^ Consequently, it was crucial to ensure that, at low concentrations, the increased steric hindrance and reduced mobility of the antioxidant within the PA nanofiber did not adversely affect its antioxidant efficacy. To examine the influence of thiol-based oxidative cross-linking on the material properties of the GSH PA nanofibers, we aimed to assess the viscosity following reduction with dithiothreitol (DTT) or oxidation with H_2_O_2_. The viscosity variations of the PA nanofiber solutions were subsequently evaluated through a steady-rate sweep test at a constant strain, where no significant change in the material’s viscosity nor the onset of the free flow plateau, was observed at shear rates greater than approximately 0.5 s^–1^ suggesting no significant measurable changes in bulk material properties (Figure S9A). At low shear rates, the more liquid like character of the DTT reduced sample was apparent from a significant shift down in measurable viscosity likely a result of DTT reducing any disulfide formation.

**Figure 4:**
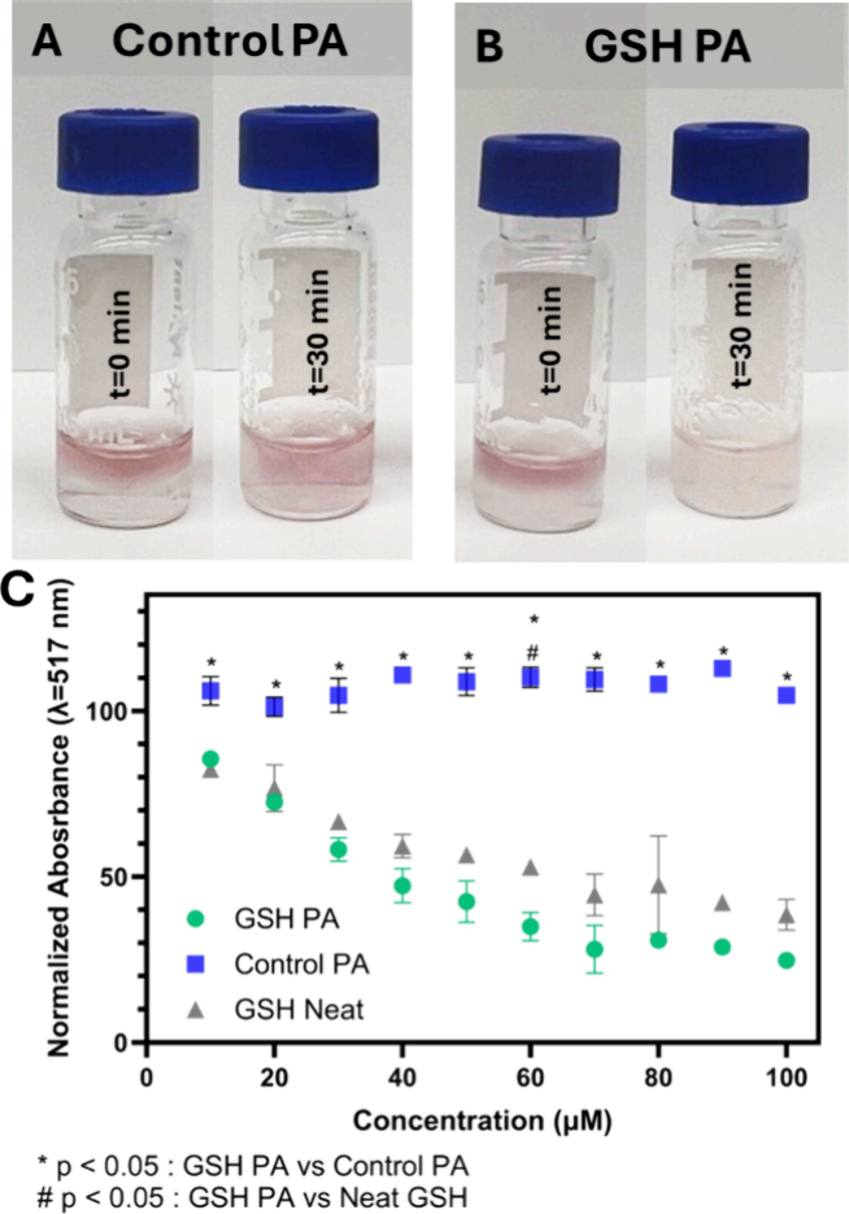
Characterization of antioxidant capabilities of PA nanofibers. (A) Gelled Control PA at 10 mM with DPPH at *t* = 0 min and following 30 min (*t* = 30 min). (B) Gelled GSH PA at 10 mM with DPPH at *t* = 0 min and following 30 min (*t* = 30 min). (C) DPPH assay comparing the radical quenching ability of the GSH PA, the Control PA, and neat glutathione (GSH). Data displayed as mean ± s.d. (*n* = 3 per treatment). One-way analysis of variance (ANOVA) with a Tukey post hoc test was used to assess significance (*p < 0.05, GSH PA vs Control PA; ^#^ *p* < 0.05, GSH PA vs Neat GSH).

Additionally, at this concentration, no functional alteration in the hydrogel was detected as a result of calcium ion cross-linking (Figure S9A). Following rheology, the samples were further analyzed in the DPPH assay to verify radical quenching capability following oxidation. At high concentrations of the GSH PA hydrogel relative to the DPPH radical, some quenching capability was maintained following the H_2_O_2_ pre-oxidation (Figure S9B). However, as anticipated, the antioxidant capability was significantly reduced following the oxidative treatment when compared to the GSH PA at comparable DPPH concentrations (Figure S9C).

To explore the potential of a hydrogel treatment, we also compared the antioxidant potential of GSH PA nanofibers pre and post ionic cross-linking with calcium ions and observed no significant differences on the antioxidant capability of the PA nanofibers (Figure S10). Ionic cross-linking with calcium is achieved through chelation of calcium with the terminal gamma-carboxyl groups of glutamic acids in the PA monomers, allowing the GSH functionality to be retained and unaffected by the ionic cross-linking (Figure S10A,B). To evaluate the maintenance of antioxidant functionality over time, gelled samples were aged on the benchtop for 1 week and incubated in the DPPH assay and compared to freshly gelled samples. We observed no visible difference in the quenching abilities of the two samples (Figure S10C), and when supernatant was collected from the gels there was no significant difference in the absorbance measured (Figure S10D). The ability for the GSH PA hydrogels to maintain antioxidant quenching capabilities at 1 week provides proof-of-principle for these biomaterials in therapeutic applications over extended periods of time. Further, these gels, consisting only of amino acids and lipids, with ionic cross-linking facilitated by physiologically safe concentrations of calcium ions,^56,57^ are considered highly biocompatible. Calcium ion content within the hydrogels was calculated to be 0.5 mol of Ca^2+^ per mole of GSH PA, with approximately 1.8 mM Ca^2+^ released from the ionic cross-linked hydrogels over a 24 h period at 37 °C (Figure S11). While forming a stable gel, the PA components are biodegradable, with no observed toxicity or immune response being reported for similar PA nanofiber materials.^58,59^ The calcium-chelated network provides structural integrity, and the calcium levels used are significantly lower than those utilized in alginate-based cross-linked systems, ensuring a gradual, physiologically safe calcium release as the gel degrades over time.^60,61^ The obtained results demonstrate the ability of the GSH PA supramolecular polymers over a wide range of concentrations to effectively scavenge DPPH radicals, indicating their antioxidant properties and ability to be used in both solution or as a hydrogel allowing for the potential broad application of these antioxidant biomaterials.

### 3.4 *In Vitro* Assessment of Antioxidant Potential of the GSH PA

With the antioxidant capabilities of the materials established on the benchtop, we then aimed to establish an *in vitro* model of oxidative damage, where cellularly produced ROS contributes directly to cellular oxidative stress and cell death. In mimicking the rise of ROS present in acute and chronic disease states, it was desired to use a compound that would be internalized by cells, contribute to the upregulation of ROS, and increase oxidative stress within the cell, ultimately resulting in the release of cellular derived damaging radicals into the extracellular environment. tert-Butyl hydroperoxide (tBHP) is an aqueous soluble compound that interferes with intercellular redox processes to increase mitochondrial ROS production.^62^ tBHP is commonly used to model oxidative damage because it activates multiple pathways that lead to ROS production. This makes it effective for studying the pathogenesis of acute ROS-induced conditions *in vitro*. Primarily, it can be metabolized into free radical intermediates that engage in lipid peroxidation and form covalent bonds with cellular molecules initiating cell damage by cytochrome P450 enzymes.^63^ Furthermore, upon cellular internalization and in the presence of GSH Peroxidase, tBHP is detoxified to tert-butanol and the free radical alters the intracellular balance of free to reduced glutathione driving ROS imbalance.^63^ Initially, to ensure that the GSH PA molecule does not chemically interact with tBHP prior to cellular internalization, the PA was coincubated with tBHP, and the DPPH radical quenching assay was performed. No change in the activity of the tBHP compared to neat GSH PA was observed by the DPPH assay, confirming no interaction between the GSH PA and tBHP was occurring (Figure S12).

tert-Butyl hydroperoxide has been shown to effectively induce ROS production that contributes to apoptosis and necroptosis in a concentration and time-dependent manner in endothelial cells.^64^ Chen et al. and Harris et al. established that incubation with this stressor for 24 h can significantly reduce cellular viability and contribute to lipid peroxidation.^64,65^ Initially, a wide range of tBHP concentrations were screened with human embryonic kidney cells (HEK 293) to determine the concentration where significant cell death begins to occur. It was crucial to achieve a substantial degree of acute tBHP induced cytotoxicity, while also maintaining a significant proportion of viable yet ROS-vulnerable cells, suitable for antioxidant rescue. Significant cell death was observed at concentrations as low as 20 μM tBHP and increased with increasing tBHP concentration as assessed by an LDH assay (Figure 5A). Additionally, this was supported by fluorescent microscopy imaging where an increase in cell death was observed with increasing tBHP concentration (Figure 5B–G). A deterioration in cellular health was evident, as indicated by the progressive loss of cell filopodia and an increase in cells displaying a more rounded morphology with increasing tBHP concentration (Figure 5B–G).

**Figure 5:**
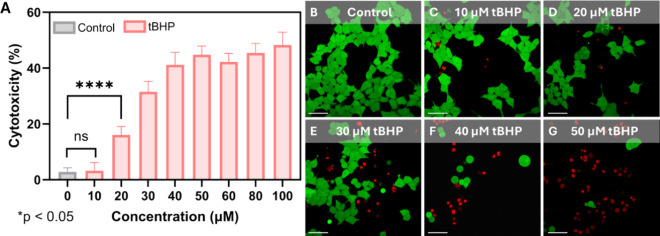
Characterization of ROS generation with the agonist tBHP. (A) Lactate dehydrogenase (LDH) assay for cytotoxicity of tBHP incubated with HEK 293 cells for 24 h. Data displayed as mean ± s.d. (*n* = 9 per treatment). One-way analysis of variance (ANOVA) with a Tukey post hoc test was used to assess significance (****p < 0.0001). Live/dead fluorescent imaging of HEK 293 cells following 24 h incubation with (B) 0 μM (Control), (C) 10 μM tBHP, (D) 20 μM tBHP, (E) 30 μM tBHP, (F) 40 μM tBHP, and (G) 50 μM tBHP. All scale bars are 50 μm.

To confirm that the observed increase in cell death and morphology changes were induced by ROS production, cells were stained with CellRox Deep Red, a fluorogenic probe used to measure oxidative stress that localizes in the cytoplasm. In control cells not treated with tBHP, no fluorescent signal was generated by the probe. However, with increasing concentration of tBHP, an increase in detected cellular oxidative stress was observed, suggesting increased ROS levels that correspond with cell death (Figure S13). Interestingly, oxidative stress was detected in samples that were incubated with 10 μM tBHP, which did not induce significant cytotoxicity at the 24 h time point. We assume that given a longer incubation period, these agitated yet vulnerable cells may experience apoptosis as a result of ROS accumulation. At higher levels of tBHP where more cell death was observed, overall cell density is reduced compared to lower concentrations of tBHP (Figure S13). A tBHP concentration of 30 μM tBHP was considered optimal as an irritant to mimic biologically relevant oxidative stress over a 24 h time frame for our *in vitro* assay to assess antioxidant activity. At a concentration of 30 μM tBHP, significant ROS production was induced, resulting in considerable oxidative stress, changes in cellular morphology, and a significant degree of cytotoxicity to the cells.

Following the development of *in vitro* assay conditions to assess cellular agitation and ROS production, we sought to evaluate the efficacy of our antioxidant GSH PA nanofibers for ROS-mitigation. PA nanofibers were prepared as dilute solutions in deionized water and added to each well 24 h after plating cells. To determine the therapeutic potential of the GSH PA as an injectable therapeutic we initially assessed the biocompatibility of the GSH PA and Control PA nanofibers *in vitro*. No significant cytotoxicity was observed following 24 h of incubation with HEK293 cells up to a concentration of 500 μM demonstrating the high level of biocompatibility of the PA nanofibers (Figure S14). To characterize the cytotoxicity of PA gel samples at higher concentrations that would be required for topical application of the gelled supramolecular polymers, a dual approach was taken. Initially, PA nanofibers at various concentrations were ionically cross-linked into hydrogels with calcium ions, followed by cells plated on top of these prepared gel samples. At 24 h, the LDH assay was performed, and was coupled with live cell imaging of the treated samples. GSH PA began to exhibit signs of increased cytotoxicity at approximately 5 mM and significant differences from the control observed at 10 mM via the LDH assay. This is consistent with previous reports that the antioxidant GSH can become toxic to cells in the millimolar concentration range,^66,67^ and it was observed that the pH of the cell culture media was altered at these high concentration incubations (Figure S15). Importantly, following 24 h of incubation, live cells were visualized moving through the matrix at all concentrations by confocal microscopy (Figure S15), highlighting the advantageous porosity of these self-assembled systems as being conducive to cell migration for regenerative applications.

To assess *in vitro* therapeutic antioxidant potential, our tBHP assay was performed assessing PA nanofiber concentrations within the range of 20–100 μM PA (Figure 6A). Control PA had no significant effect on the observed cytotoxicity at all concentrations tested, however, the GSH PA nanofibers significantly lowered the cellular cytotoxicity at every concentration investigated (Figure 6A). This observation was further supported by fluorescence microscopy which demonstrated a marked improvement in cellular morphology following incubation with the antioxidant GSH PA nanofibers, accompanied by a significant reduction in the number of observed dead cells (Figure 6B–E). There was no significant difference between the Control PA nanofiber treated and the tBHP only control, confirming that, as expected, without the glutathione antioxidant moiety incorporated, no ROS-rescue of cells was observed (Figure 6F–I).

**Figure 6:**
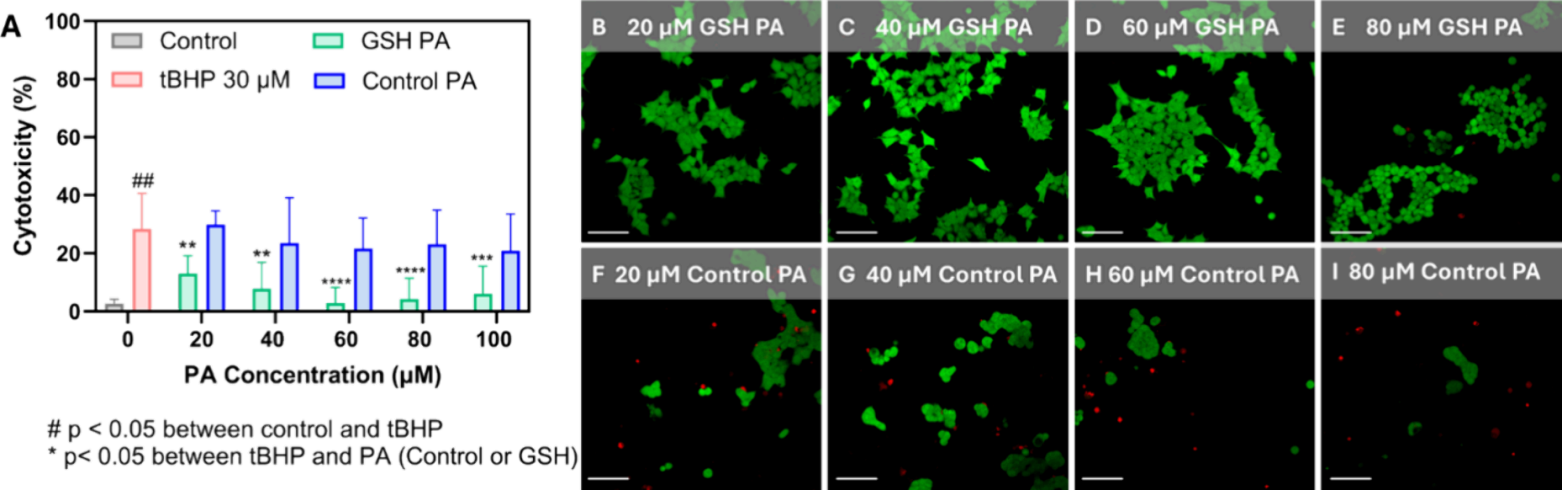
Characterization of cell rescue via antioxidant GSH PA nanofibers. (A) Lactate dehydrogenase (LDH) assay for cytotoxicity of tBHP incubated with HEK 293 cells for 24 h with PA nanofiber treatments. Data displayed as mean ± s.d. (*n* = 9 per treatment). One-way analysis of variance (ANOVA) with a Tukey post hoc test to assess significance (^#^ indicates significance in comparison between control and 30 μM tBHP treated, while * compares the PA nanofiber treatments to 30 μM tBHP treated). Live/dead fluorescent imaging of HEK 293 cells following 24 h incubation with 30 μM tBHP and (B) 20 μM GSH PA, (C) 40 μM GSH PA, (D) 60 μM GSH PA, (E) 80 μM GSH PA. Live/dead fluorescent imaging of HEK 293 cells following 24 h incubation with 30 μM tBHP and (F) 20 μM Control PA, (G) 40 μM Control PA, (H) 60 μM Control PA, (I) 80 μM Control PA. All scale bars are 50 μm.

Interestingly, assessment of neat GSH in the tBHP assay required concentrations of 1 mM or higher to achieve significant cell rescue (Figure S16), an order of magnitude higher than the antioxidant supramolecular polymers (Figure 6A). Molecular GSH when added to cell culture media is internalized by cells and interferes with the balance of intracellular redox pathways.^68^ Our GSH PA nanofibers were designed to be sufficiently large enough to remain in the extracellular space, and in turn provide a localized mitigation of extracellular ROS. We believe this enhanced efficacy is a direct result of the role the supramolecular polymer plays in increasing the proximal concentration of GSH molecules within the polymer compared to neat GSH, and maintaining this concentration in the extracellular region, effectively mitigating secondary degeneration through extracellular ROS transfer between cells. PA nanofibers are commonly used in extracellular applications,^33,39,40,44,52^ where the lack of a cell-penetrating epitope, and the fibrous nature of the PA strongly reduce the likelihood of significant cellular internalization.^69,70^ However, cellular uptake of the PA nanofibers was not directly studied in this work.

Finally, the CellRox Deep Red fluorescent probe was used to quantify oxidative stress following exposure to tBHP and the PA nanofibers. Following previously established image analysis protocols,^48^ the number of CellRox positive pixels was quantified and then normalized by the number of cell nuclei in the frame per treatment. Following this analysis, it was observed that the treatment of cells with the antioxidant GSH PA nanofibers served to significantly reduce the oxidative stress present in the cells (Figure 7A). Additionally, an increase in oxidative stress was observed with fluorescent confocal microscopy, where GSH PA treated cells displayed minimal CellRox positive puncta compared to the Control PA treated cells consistently being CellRox positive (Figure 7B–G). Although there were still cells that were stressed by the tBHP with the GSH PA treatment (Figure S17), the overall count of pixels that were positive was significantly reduced when compared to the tBHP-only treated samples. In these images, it was more common for a single cell to be strongly agitated while surrounding cells appeared healthy, compared to the Control PA and tBHP samples which had most cells displaying signs of significant oxidative stress and morphology abnormalities compared to no treatment controls.

**Figure 7:**
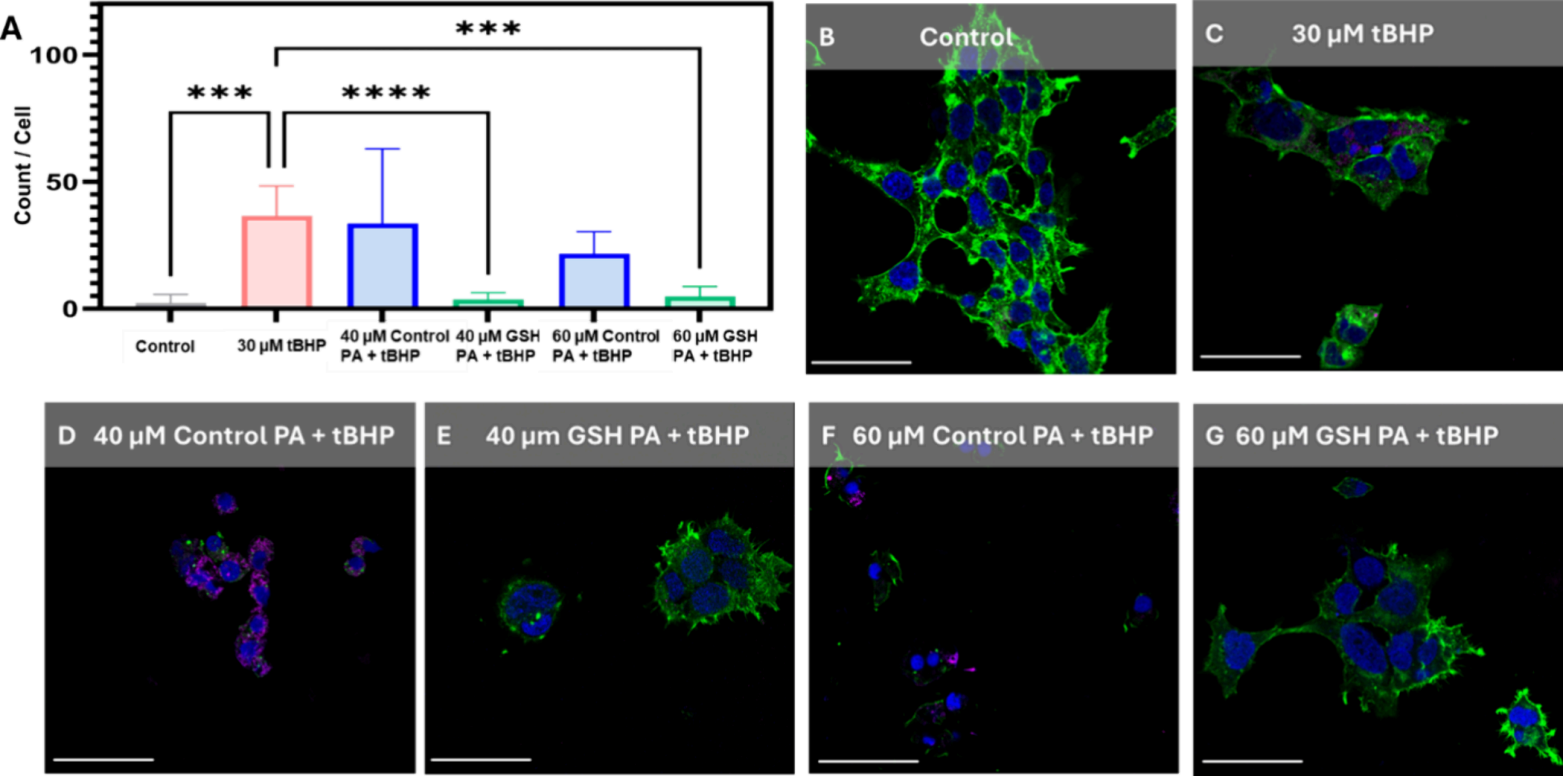
Characterization of the oxidative stress of cells treated with tBHP and PA nanofibers. (A) Analysis of oxidative stress per cell following treatments. Data displayed as mean ± s.d. (*n* = 9 images, minimum of 275 cells analyzed at 20× magnification per treatment). One-way analysis of variance (ANOVA) with a Tukey post hoc test was used to assess significance (****p < 0.0001). Representative fluorescent confocal micrographs with labeled nuclei (DAPI, blue), actin (Phalloidin iFluor Actin Stain, green) and oxidative stress (CellRox, magenta) of (B) no treatment control cells, (C) 30 μM tBHP, (D) 30 μM tBHP + 40 μM Control PA, (E) 30 μM tBHP + 40 μM GSH PA, (F) 30 μM tBHP + 60 μM Control PA, (G) 30 μM tBHP + 60 μM GSH PA. All micrograph scale bars are 50 μm.

## 4 Conclusions

Herein we have developed an antioxidant peptide amphiphile (GSH PA) that maintains the activity of glutathione when tethered to a supramolecular polymer. The ability to self-assemble into nanofibers was relatively unaffected by the addition of the bioactive glutathione although shorter nanofibers were observed for the GSH PA relative to the Control PA, an observation likely a result of enhanced solubility of the GSH PA relative to the Control PA monomers at physiological pH. We further explored the ability to work with these materials in a solution state or as a physical hydrogel, with antioxidant activity retained in a physical, ionically cross-linked PA nanofiber network as well as at low concentrations in solution. Importantly, the GSH PA nanofibers achieved antioxidant cell rescue at concentrations an order of magnitude lower than molecular glutathione, a result related to both the extracellular localization, and enhancement in proximal concentration of GSH moieties along the supramolecular polymer. This study demonstrates the potential of GSH PA-based nanofibers as a versatile platform for combating oxidative stress. The GSH PA nanofibers can be localized within the extracellular space acting as a scavenger that can mitigate the oxidative burst and secondary degeneration caused by damaging ROS propagation.

The ability to tune the material’s viscosity and transition between solution and gel states opens the door for a wide range of applications, from injectable therapeutics to topically applied hydrogels. This flexibility is particularly promising for accelerating tissue regeneration in injury or disease states highly affected by ROS. In recent work, similar supramolecular polymers demonstrated good tissue adhesion to the surface of organs,^71^ suggesting the ability to topically apply GSH PA supramolecular polymers with good adherence also. Similarly therapeutic targeting of PA nanofibers has also been achieved by incorporating peptide sequences to direct similar materials toward specific disease indications.^33,41,42,72^ While not explored in this study, these previous works demonstrate a clear potential for integrating peptide targeting sequences, to enhance the precision and efficacy of our antioxidant platform. This approach has the ability to mitigate ROS-driven damage, provide a scaffold to promote regeneration, and provides a versatile antioxidant therapy that could be applied across a range of diseases and injuries where oxidative stress plays a critical role in disease progression and tissue damage.

## Supplementary Material







## Notes

Phantom injection of the Control PA hydrogel into a chicken drumstick (MP4)
Phantom injection of the GSH PA hydrogel into a chicken drumstick (MP4)
Peptide amphiphile characterization including LC-MS analysis; ^13^C and ^1^H-NMR; critical aggregation concentration; additional rheology; SEM characterization; DPPH studies; and cytotoxicity assessments (PDF)

## References

[1] Alfadda A. A., Sallam R. M. (2012). Reactive oxygen
species in health and disease. J. Biomed. Biotechnol..

[2] Droge W. (2002). Free radicals
in the physiological control of cell function. Physiol Rev..

[3] Tsao C. W., Aday A. W., Almarzooq Z. I., Anderson C. A. M., Arora P., Avery C. L., Baker-Smith C. M., Beaton A. Z., Boehme A. K., Buxton A. E. (2023). Heart
Disease and Stroke Statistics—2023
Update: A Report From the American Heart Association. Circulation.

[4] Gerstl J. V. E., Ehsan A. N., Lassarén P., Yearley A., Raykar N. P., Anderson G. A., Smith T. R., Sabapathy S. R., Ranganathan K. (2024). The Global Macroeconomic Burden of
Burn Injuries. Plast Reconstr Surg.

[5] Reddy V. P. (2023). Oxidative
Stress in Health and Disease. Biomedicines.

[6] Burtenshaw D., Kitching M., Redmond E. M., Megson I. L., Cahill P. A. (2019). Reactive
Oxygen Species (ROS), Intimal Thickening, and Subclinical Atherosclerotic
Disease. Front Cardiovasc Med..

[7] Cadenas S. (2018). ROS and redox
signaling in myocardial ischemia-reperfusion injury and cardioprotection. Free Radic Biol. Med..

[8] Kawagishi H., Finkel T. (2014). Unraveling the truth
about antioxidants: ROS and disease:
finding the right balance. Nat. Med..

[9] Jia Z., Zhu H., Li J., Wang X., Misra H., Li Y. (2012). Oxidative
stress in spinal cord injury and antioxidant-based intervention. Spinal Cord.

[10] Lin M. T., Beal M. F. (2006). Mitochondrial dysfunction and oxidative stress in neurodegenerative
diseases. Nature.

[11] Nita M., Grzybowski A. (2016). The Role of
the Reactive Oxygen Species and Oxidative
Stress in the Pathomechanism of the Age-Related Ocular Diseases and
Other Pathologies of the Anterior and Posterior Eye Segments in Adults. Oxid Med. Cell Longev.

[12] Hardy N., Viola H. M., Johnstone V. P., Clemons T. D., Cserne
Szappanos H., Singh R., Smith N. M., Iyer K. S., Hool L. C. (2015). Nanoparticle-mediated dual delivery of an antioxidant
and a peptide against the L-Type Ca2+ channel enables simultaneous
reduction of cardiac ischemia-reperfusion injury. ACS Nano.

[13] Li C.-W., Li L.-L., Chen S., Zhang J.-X., Lu W.-L. (2020). Antioxidant
Nanotherapies for the Treatment of Inflammatory Diseases. Front. Bioeng. Biotechnol..

[14] Firuzi O., Miri R., Tavakkoli M., Saso L. (2011). Antioxidant therapy:
current status and future prospects. Curr. Med.
Chem..

[15] Benfeito S., Oliveira C., Soares P., Fernandes C., Silva T., Teixeira J., Borges F. (2013). Antioxidant therapy:
Still in search of the ‘magic bullet’. Strategies to target mitochondria and oxidative stress.

[16] Li W., Yang S. (2016). Targeting
oxidative stress for the treatment of ischemic stroke:
Upstream and downstream therapeutic strategies. Brain Circ.

[17] Loor G., Kondapalli J., Iwase H., Chandel N. S., Waypa G. B., Guzy R. D., Vanden Hoek T. L., Schumacker P. T. (2011). Mitochondrial
oxidant stress triggers cell death in simulated ischemia-reperfusion. Biochim. Biophys. Acta.

[18] Rodrigo R., Libuy M., Feliu F., Hasson D. (2013). Molecular basis of
cardioprotective effect of antioxidant vitamins in myocardial infarction. Biomed. Res. Int..

[19] De
Ceaurriz J., Payan J. P., Morel G., Brondeau M. T. (1994). Role of
extracellular glutathione and γ-Glutamyltranspeptidase in the
disposition and kidney toxicity of inorganic mercury in rats. Journal of Applied Toxicology.

[20] Jahan U. M., Blevins B., Minko S., Reukov V. V. (2025). Advancing biomedical
applications: antioxidant and biocompatible cerium oxide nanoparticle-integrated
poly-ε-caprolactone fibers. Biomed. Phys.
Eng. Express.

[21] Yang B., Chen Y., Shi J. (2019). Reactive Oxygen Species
(ROS)-Based
Nanomedicine. Chem. Rev..

[22] Yang P., Gu Z., Zhu F., Li Y. (2020). Structural and Functional Tailoring
of Melanin-Like Polydopamine Radical Scavengers. CCS Chemistry.

[23] Xu Y., Hu J., Hu J., Cheng Y., Chen X., Gu Z., Li Y. (2023). Bioinspired
polydopamine hydrogels: Strategies and applications. Prog. Polym. Sci..

[24] Cao H., Yang L., Tian R., Wu H., Gu Z., Li Y. (2022). Versatile polyphenolic platforms
in regulating cell biology. Chem. Soc. Rev..

[25] McCallum N.
C., Son F. A., Clemons T. D., Weigand S. J., Gnanasekaran K., Battistella C., Barnes B. E., Abeyratne-Perera H., Siwicka Z. E., Forman C. J. (2021). Allomelanin: A Biopolymer
of Intrinsic Microporosity. J. Am. Chem. Soc..

[26] Biyashev D., Siwicka Z. E., Onay U. V., Demczuk M., Xu D., Ernst M. K., Evans S. T., Nguyen C. V., Son F. A., Paul N. K. (2023). Topical application of synthetic melanin promotes tissue
repair. NPJ. Regen Med..

[27] Pu Y., Wang P., Yang R., Tan X., Shi T., Ma J., Xue W., Chi B. (2022). Bio-fabricated
nanocomposite hydrogel
with ROS scavenging and local oxygenation accelerates diabetic wound
healing. J. Mater. Chem. B.

[28] Li Z., Zhao Y., Huang H., Zhang C., Liu H., Wang Z., Yi M., Xie N., Shen Y., Ren X. (2022). A Nanozyme-Immobilized
Hydrogel with Endogenous ROS-Scavenging
and Oxygen Generation Abilities for Significantly Promoting Oxidative
Diabetic Wound Healing. Adv. Healthcare Mater..

[29] Xu Q., He C., Xiao C., Chen X. (2016). Reactive Oxygen Species (ROS) Responsive
Polymers for Biomedical Applications. Macromol.
Biosci.

[30] Ye H., Zhou Y., Liu X., Chen Y., Duan S., Zhu R., Liu Y., Yin L. (2019). Recent Advances on Reactive Oxygen
Species-Responsive Delivery and Diagnosis System. Biomacromolecules.

[31] Hara Y., Yoshizawa K., Yaguchi A., Hiramatsu H., Uchida N., Muraoka T. (2024). ROS-Responsive
Methionine-Containing
Amphiphilic Peptides Impart Enzyme-Triggered Phase Transition and
Antioxidant Cell Protection. Biomacromolecules.

[32] Hussain M., Suo H., Xie Y., Wang K., Wang H., Hou Z., Gao Y., Zhang L., Tao J., Jiang H. (2021). Dopamine-Substituted
Multidomain Peptide Hydrogel with Inherent Antimicrobial Activity
and Antioxidant Capability for Infected Wound Healing. ACS Appl. Mater. Interfaces.

[33] Peters E. B., Karver M. R., Sun K., Gillis D. C., Biswas S., Clemons T. D., He W., Tsihlis N. D., Stupp S. I., Kibbe M. R. (2021). Self-Assembled Peptide
Amphiphile Nanofibers for Controlled
Therapeutic Delivery to the Atherosclerotic Niche. Advanced Therapeutics.

[34] Wu Y., Wang Y., Long L., Hu C., Kong Q., Wang Y. (2022). A spatiotemporal release platform based on pH/ROS stimuli-responsive
hydrogel in wound repairing. J. Controlled Release.

[35] Stupp S. I., Clemons T. D., Carrow J. K., Sai H., Palmer L. C. (2020). Supramolecular
and Hybrid Bonding Polymers. Isr J. Chem..

[36] Webber M. J., Tongers J., Newcomb C. J., Marquardt K. T., Bauersachs J., Losordo D. W., Stupp S. I. (2011). Supramolecular nanostructures
that mimic VEGF as a strategy for ischemic tissue repair. P Natl. Acad. Sci. USA.

[37] Sather N. A., Sai H., Sasselli I. R., Sato K., Ji W., Synatschke C. V., Zambrotta R. T., Edelbrock J. F., Kohlmeyer R. R., Hardin J. O. (2021). 3D Printing of Supramolecular Polymer Hydrogels
with Hierarchical Structure. Small.

[38] Edelbrock A. N., Clemons T. D., Chin S. M., Roan J. J. W., Bruckner E. P., Alvarez Z., Edelbrock J., Wek K. S., Stupp S. I. (2021). Superstructured
Biomaterials Formed by Exchange Dynamics and Host-Guest Interactions
in Supramolecular Polymers. Adv. Sci..

[39] Klein M. K., Kassam H. A., Lee R. H., Bergmeier W., Peters E. B., Gillis D. C., Dandurand B. R., Rouan J. R., Karver M. R., Struble M. D. (2020). Development
of Optimized Tissue-Factor-Targeted Peptide Amphiphile Nanofibers
to Slow Noncompressible Torso Hemorrhage. ACS
Nano.

[40] Ledford B. T., Akerman A. W., Sun K., Gillis D. C., Weiss J. M., Vang J., Willcox S., Clemons T. D., Sai H., Qiu R. (2022). Peptide Amphiphile Supramolecular Nanofibers Designed
to Target Abdominal Aortic Aneurysms. ACS Nano.

[41] Mansukhani N. A., Peters E. B., So M. M., Albaghdadi M. S., Wang Z., Karver M. R., Clemons T. D., Laux J. P., Tsihlis N. D., Stupp S. I. (2019). Peptide
Amphiphile Supramolecular
Nanostructures as a Targeted Therapy for Atherosclerosis. Macromol. Biosci.

[42] Marulanda K., Mercel A., Gillis D. C., Sun K., Gambarian M., Roark J., Weiss J., Tsihlis N. D., Karver M. R., Centeno S. R. (2021). Intravenous Delivery
of Lung-Targeted Nanofibers
for Pulmonary Hypertension in Mice. Adv. Healthc
Mater..

[43] Lee S., Carrow J. K., Fraser L. A., Yan J., Jeyamogan S., Sambandam Y., Clemons T. D., Kolberg-Edelbrock A. N., He J., Mathew J. (2024). Single-cell coating with biomimetic extracellular
nanofiber matrices. Acta Biomater.

[44] Lewis J. A., Freeman R., Carrow J. K., Clemons T. D., Palmer L. C., Stupp S. I. (2020). Transforming Growth
Facto beta-1 Binding by Peptide
Amphiphile Hydrogels. Acs Biomater Sci. Eng..

[45] Smith C. S., Alvarez Z., Qiu R., Sasselli I. R., Clemons T., Ortega J. A., Vilela-Picos M., Wellman H., Kiskinis E., Stupp S. I. (2023). Enhanced Neuron Growth and Electrical Activity by a
Supramolecular Netrin-1 Mimetic Nanofiber. ACS
Nano.

[46] Abernathy H. G., Saha J., Kemp L. K., Wadhwani P., Clemons T. D., Morgan S. E., Rangachari V. (2023). De novo amyloid
peptides with subtle
sequence variations differ in their self-assembly and nanomechanical
properties. Soft Matter.

[47] Mondal M., Jankoski P. E., Lee L. D., Dinakarapandian D. M., Chiu T. Y., Swetman W. S., Wu H., Paravastu A. K., Clemons T. D., Rangachari V. (2024). Reversible
Disulfide Bond Cross-Links
as Tunable Levers of Phase Separation in Designer Biomolecular Condensates. J. Am. Chem. Soc..

[48] Shihan M. H., Novo S. G., Le Marchand S. J., Wang Y., Duncan M. K. (2021). A simple
method for quantitating confocal fluorescent images. Biochem. Biophys. Rep..

[49] Forman H. J., Zhang H., Rinna A. (2009). Glutathione:
overview of its protective
roles, measurement, and biosynthesis. Mol. Aspects
Med..

[50] Yin W., Ke W. D., Lu N. N., Wang Y. H., Japir A. W. M. M., Mohammed F., Wang Y., Pan Y. Y., Ge Z. S. (2020). Glutathione
and Reactive Oxygen Species Dual-Responsive Block Copolymer Prodrugs
for Boosting Tumor Site-Specific Drug Release and Enhanced Antitumor
Efficacy. Biomacromolecules.

[51] Clemons T. D., Egner S. A., Grzybek J., Roan J. J., Sai H., Yang Y., Syrgiannis Z., Sun H., Palmer L. C., Gianneschi N. C. (2024). Hybrid Bonding Bottlebrush
Polymers Grafted
from a Supramolecular Polymer Backbone. J. Am.
Chem. Soc..

[52] Qiu R., Chen F., Alvarez Z., Clemons T. D., Biswas S., Karver M. R., Takata N., Sai H., Peng H., Weigand S. (2023). Supramolecular Nanofibers Block SARS-CoV-2 Entry into
Human Host Cells. ACS Appl. Mater. Interfaces.

[53] Freeman R., Han M., Alvarez Z., Lewis J. A., Wester J. R., Stephanopoulos N., McClendon M. T., Lynsky C., Godbe J. M., Sangji H. (2018). Reversible self-assembly of superstructured networks. Science.

[54] Uman S., Dhand A., Burdick J. A. (2020). Recent
advances in shear-thinning
and self-healing hydrogels for biomedical applications. J. Appl. Polym. Sci..

[55] Lowe C. J., DiMartini E. T., Mirmajlesi K. R., Gormley A. J., Shreiber D. I. (2019). Free radical-mediated
targeting and immobilization of coupled payloads. J. Drug Target.

[56] Li J., Wu Y., He J., Huang Y. (2016). A new insight to the effect of calcium
concentration on gelation process and physical properties of alginate
films. J. Mater. Sci..

[57] Li X., Liu T., Song K., Yao L., Ge D., Bao C., Ma X., Cui Z. (2006). Culture of
Neural Stem Cells in Calcium Alginate Beads. Biotechnol. Prog..

[58] Ghanaati S., Webber M. J., Unger R. E., Orth C., Hulvat J. F., Kiehna S. E., Barbeck M., Rasic A., Stupp S. I., Kirkpatrick C. J. (2009). Dynamic
in vivo biocompatibility of angiogenic peptide
amphiphile nanofibers. Biomaterials.

[59] Zhou S., Hokugo A., McClendon M., Zhang Z., Bakshi R., Wang L., Segovia L. A., Rezzadeh K., Stupp S. I., Jarrahy R. (2019). Bioactive peptide amphiphile nanofiber gels enhance
burn wound healing. Burns.

[60] Dodero A., Pianella L., Vicini S., Alloisio M., Ottonelli M., Castellano M. (2019). Alginate-based
hydrogels prepared via ionic gelation:
An experimental design approach to predict the crosslinking degree. Eur. Polym. J..

[61] Bajpai S. K., Sharma S. (2004). Investigation of swelling/degradation
behaviour of
alginate beads crosslinked with Ca2+ and Ba2+ ions. React. Funct. Polym..

[62] Zhao K., Luo G., Giannelli S., Szeto H. H. (2005). Mitochondria-targeted peptide prevents
mitochondrial depolarization and apoptosis induced by tert-butyl hydroperoxide
in neuronal cell lines. Biochem. Pharmacol..

[63] Kucera O., Endlicher R., Rousar T., Lotkova H., Garnol T., Drahota Z., Cervinkova Z. (2014). The effect of tert-butyl hydroperoxide-induced
oxidative stress on lean and steatotic rat hepatocytes in vitro. Oxid. Med. Cell Longevity.

[64] Zhao W., Feng H., Sun W., Liu K., Lu J. J., Chen X. (2017). Tert-butyl hydroperoxide (t-BHP) induced apoptosis and necroptosis
in endothelial cells: Roles of NOX4 and mitochondrion. Redox Biol..

[65] Loch-Caruso R., Korte C. S., Hogan K. A., Liao S., Harris C. (2020). Tert-Butyl
Hydroperoxide Stimulated Apoptosis Independent of Prostaglandin E(2)
and IL-6 in the HTR-8/SVneo Human Placental Cell Line. Reprod Sci..

[66] Zhang H., Limphong P., Pieper J., Liu Q., Rodesch C. K., Christians E., Benjamin I. J. (2012). Glutathione-dependent
reductive stress
triggers mitochondrial oxidation and cytotoxicity. FASEB J..

[67] Xiao W., Loscalzo J. (2020). Metabolic Responses
to Reductive Stress. Antioxid Redox Signal.

[68] Lushchak V. I. (2012). Glutathione
homeostasis and functions: potential targets for medical interventions. J. Amino Acids.

[69] Mazumdar S., Chitkara D., Mittal A. (2021). Exploration
and insights into the
cellular internalization and intracellular fate of amphiphilic polymeric
nanocarriers. Acta Pharm. Sin. B.

[70] Yameen B., Choi W. I., Vilos C., Swami A., Shi J., Farokhzad O. C. (2014). Insight
into nanoparticle cellular uptake and intracellular
targeting. 30th Anniversary Special Issue.

[71] Anderson C. F., Chakroun R. W., Grimmett M. E., Domalewski C. J., Wang F., Cui H. (2022). Collagen-Binding Peptide-Enabled
Supramolecular Hydrogel Design for Improved Organ Adhesion and Sprayable
Therapeutic Delivery. Nano Lett..

[72] Mercel A. I., Marulanda K., Gillis D. C., Sun K., Clemons T. D., Willcox S., Griffith J., Peters E. B., Karver M. R., Tsihlis N. D. (2021). Development of novel nanofibers targeted to
smoke-injured lungs. Biomaterials.

