# Supplementary material for: Combating Reactive Oxygen Species (ROS) with Antioxidant Supramolecular Polymers
Source: ACS Appl Mater Interfaces. 2025 Jun 4;17(24):35275–87. doi: 10.1021/acsami.5c06967 (PMC12186237; doi:10.1021/acsami.5c06967)
Supplement: Supplementary file 3 [file am5c06967_si_003.pdf]

Supporting Information

Combating Reactive Oxygen Species (ROS) with Antioxidant Supramolecular Polymers

Penelope E. Jankoski,[1] Zacchaeus M. Wallace,[1] Loria R. DiMartino,[1] Jessica Shrestha,[1] Ashe M. Davis,[1] Iyanuoluwani Owolabi,[2,3] Alex S. Flynt,[4] and Tristan D. Clemons.[1,2*]

1 School of Polymer Science and Engineering, University of Southern Mississippi, Hattiesburg, MS, 39406, USA.
2 Center for Molecular and Cellular Biosciences, University of Southern Mississippi, Hattiesburg, Mississippi 39406, USA.
3 School of Biological, Environmental, and Earth Sciences, University of Southern Mississippi, Hattiesburg, Mississippi 39406, USA.
4 Department of Biomedical Engineering, University of Mississippi, Oxford, Mississippi, 38677, USA.

*Correspondence should be addressed to T.D.C (email: Tristan.clemons@usm.edu)

Contents

Supporting Figures 2
- Figure S1. LC-MS analysis of Control PA. 2
- Figure S2. LC-MS analysis of GSH PA. 3
- Figure S3. ^{13}C and ^{1}H-NMR (600 MHz, TFA-d1) Control PA. 4
- Figure S4. ^{13}C and ^{1}H-NMR (600 MHz, TFA-d1) of GSH PA. 5
- Figure S5. Critical aggregate concentration determination by Nile Red. 6
- Figure S6. Steady Shear rate sweep of low concentration PA nanofibers. 7
- Figure S7. Scanning electron microscopy (SEM) characterization with ionic crosslinking. 8
- Figure S8. Validation of injectability of the GSH and Control PA into a chicken drumstick. 9
- Figure S9. Material properties following oxidation and reduction with H_2O_2/ DTT. 10
- Figure S10. DPPH studies of gelled PAs. 11
- Figure S11. Quantification of Ca^{2+} release from the GSH PA hydrogel. 12
- Figure S12. DPPH assay of PAs co-incubated with tBHP. 13
- Figure S13. tBHP concentration screening for influence on cellular oxidative stress 14
- Figure S14. Cytotoxicity of GSH PA and Control PA. 15
- Figure S15. Cytotoxicity of high concentration gelled GSH PA and Control PA. 16
- Figure S16. Understanding the impacts of untethered GSH in a tBHP ROS system 17
- Figure S17. Quantification and representative images used for oxidative stress assessment 18

Supporting Video Captions 19
- Video S1. Phantom injection of the Control PA hydrogel into a chicken drumstick. 19
- Video S2. Phantom injection of the GSH PA hydrogel into a chicken drumstick. 19

Supporting Information References 19

Supporting Figures

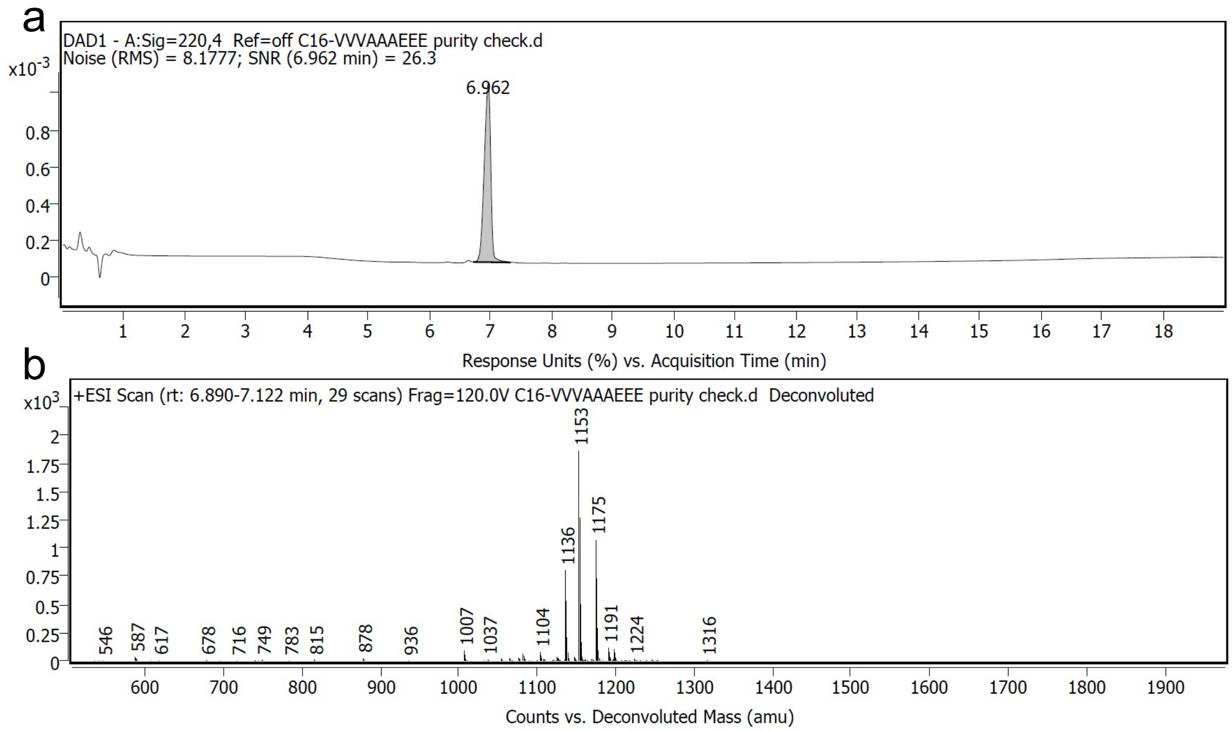

Figure S1. LC-MS analysis of Control PA. a) LC-MS trace of control PA [PA] = 1 mg/mL, loading solvent; H_2O with 0.1% NH_4OH (v/v), eluent; H_2O-CH_3CN gradient containing 0.1% HCOOH (v/v), column; Phenomenex Gemini 5 µm C18 110 Å LC column 150 x 1 mm and b) ESI-mass spectra corresponding to elution time 6.89 – 7.12 min.

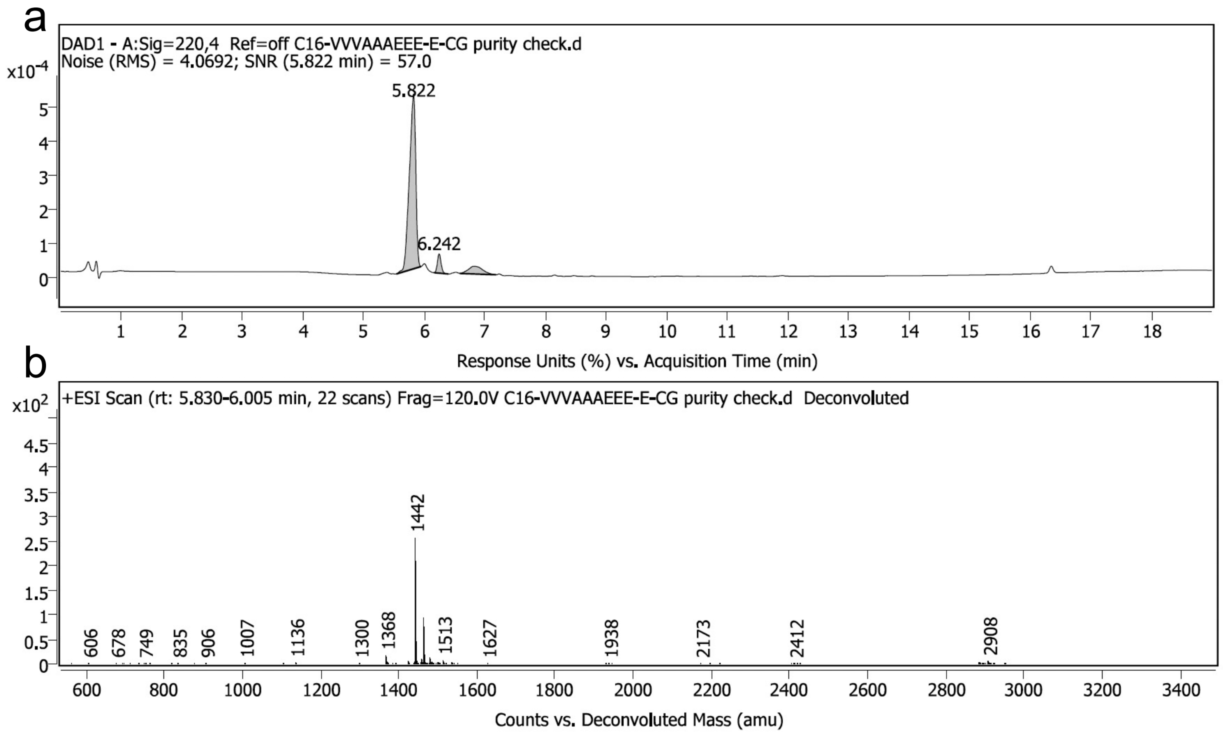

Figure S2. LC-MS analysis of GSH PA. a) LC-MS trace of GSH PA [PA] = 1 mg/mL, loading solvent; H_2O with 0.1% NH_4OH (v/v), eluent; H_2O-CH_3CN gradient containing 0.1% HCOOH (v/v), column; Phenomenex Gemini 5 µm C18 110 Å LC column 150 x 1 mm and b)ESI-mass spectra corresponding to elution time 5.83 – 6.01 min.

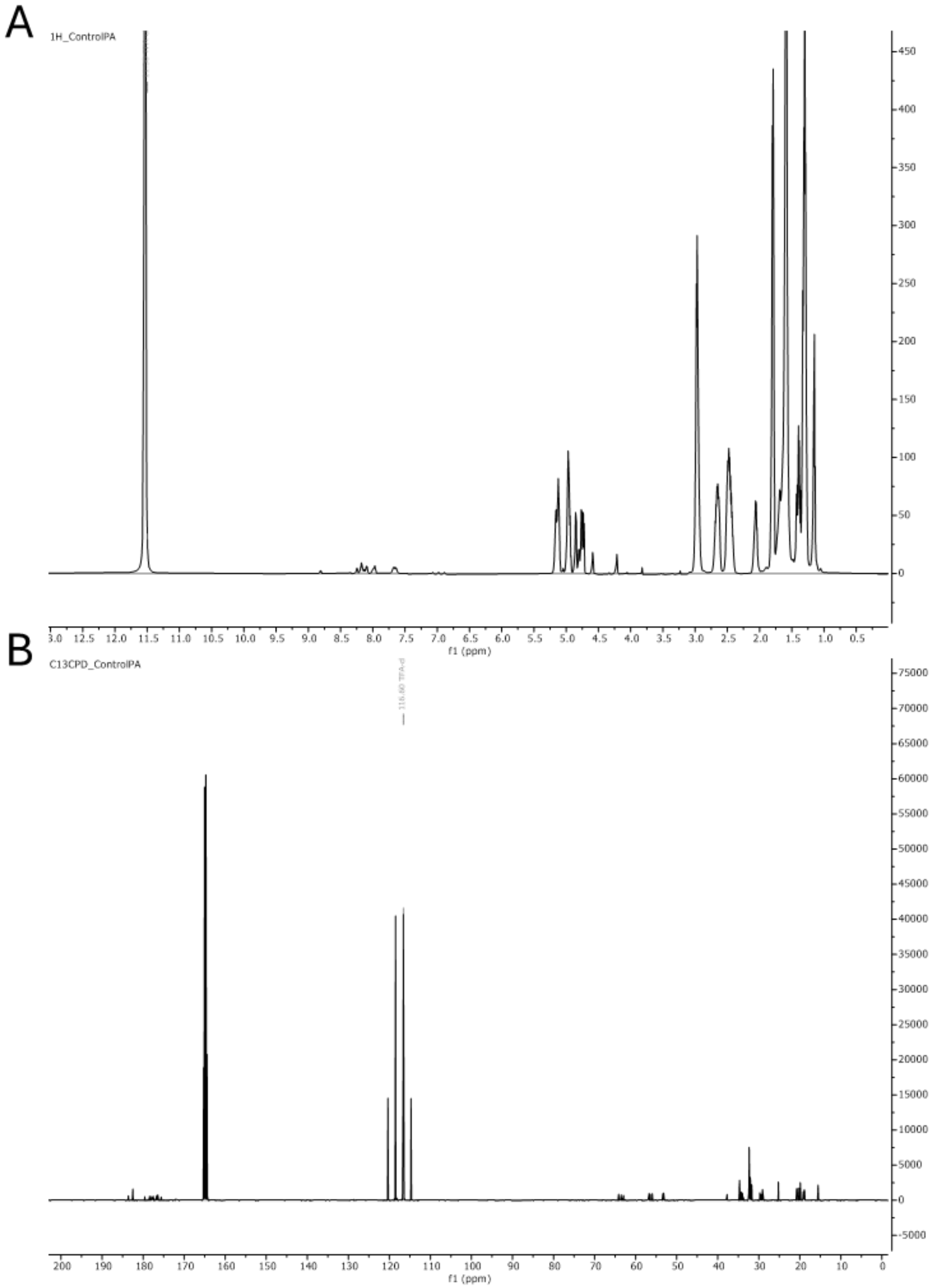

Figure S3. ^{13}C and ^{1}H-NMR (600 MHz, TFA-d1) Control PA. A) Proton NMR of Control PA confirming synthesis of molecule. B) Carbon spectra confirming synthesis, aligns with published literature on this molecule. See source for assignments.[1]

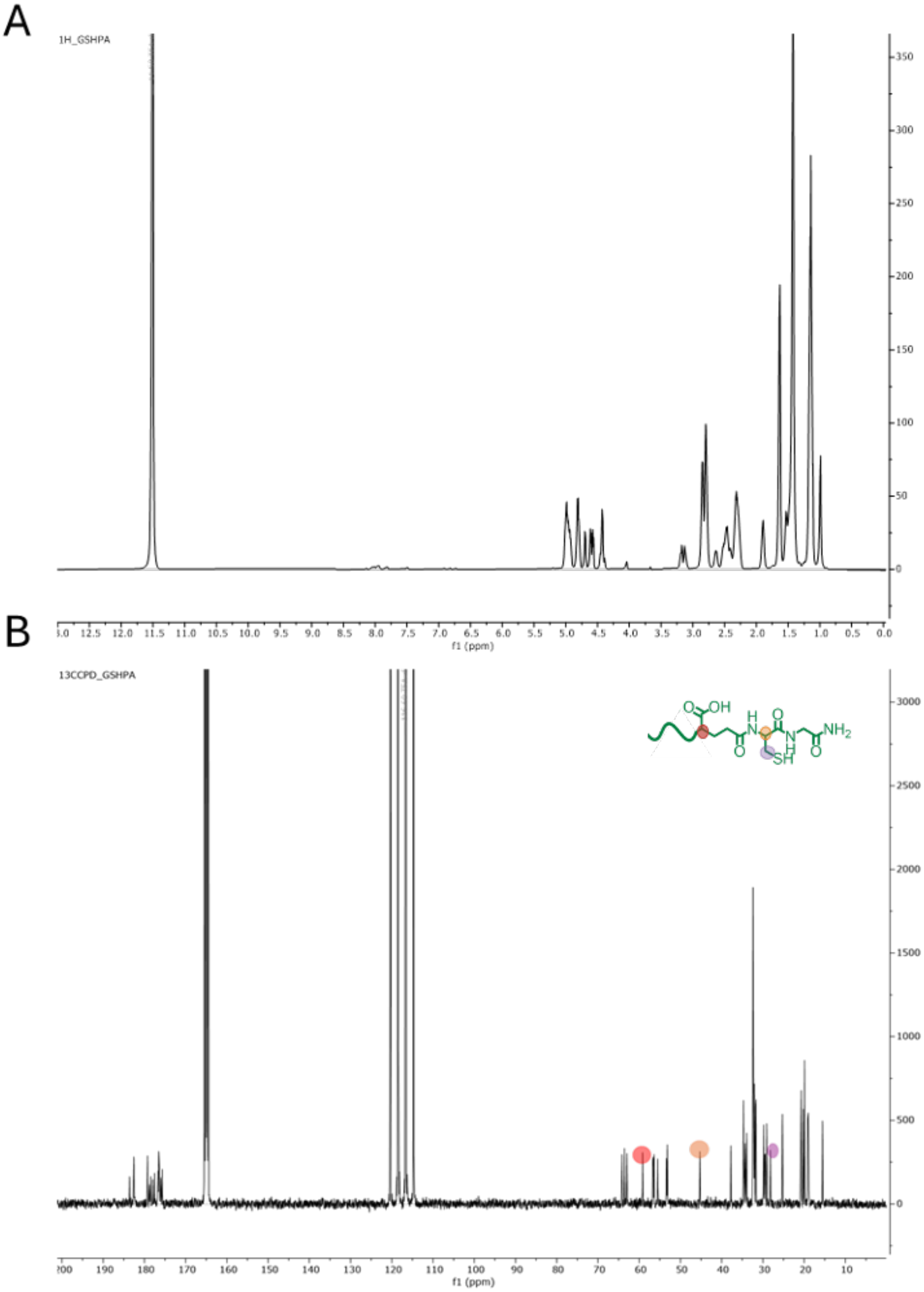

Figure S4. ^{13}C and ^{1}H-NMR (600 MHz, TFA-d1) of GSH PA. A) Proton NMR of GSH PA. Chemical shifts move to lower ppm compared to the control PA as a result of the thiol incorporation. B) Carbon spectra showing three new peaks when compared to the Control PA, denoted by red, orange, and purple highlights on the spectra, as a result of extending the control PA with the tripeptide sequence glu-cys-gly with a gamma peptide linkage between the carboxylic acid of the glutamic acid side chain and the cysteine residue to accurately mimic glutathione. See source for control PA assignments and comparison.[1]

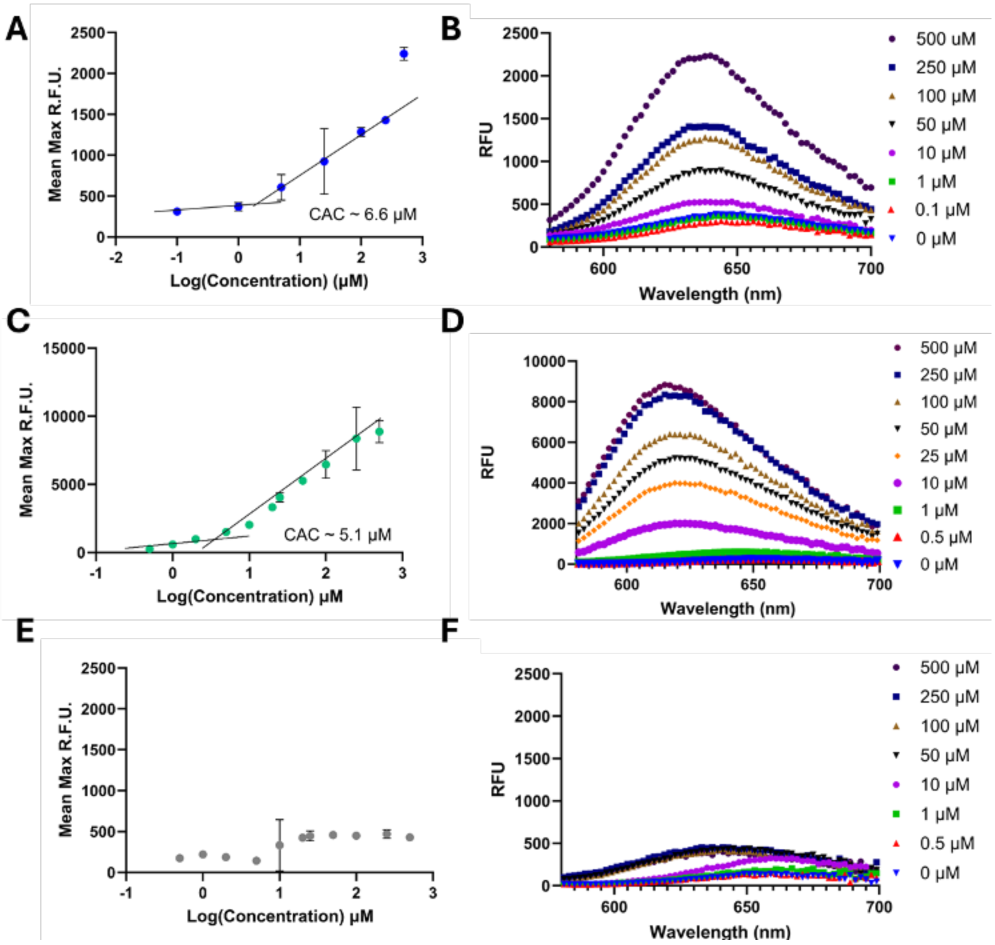

Figure S5. Critical aggregate concentration determination by Nile Red. A) Control PA CAC graph and B) spectral scans which were used to calculate the CAC for the Control PA to be 6.6 µM. C) GSH PA CAC graph and D) spectral scans which were used to calculate the CAC for the GSH PA to be 5.1 µM. E) Neat GSH CAC graph and F) spectral scans for neat GSH indicating no appreciable Nile Red fluorescence observed due to no molecular aggregation achieved with this sample.

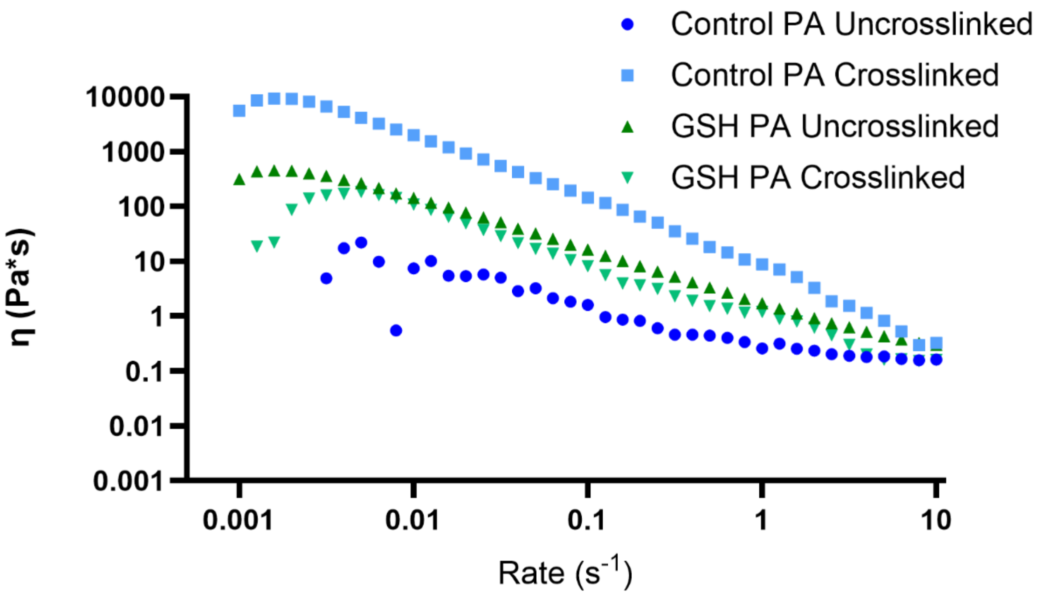

Figure S6. Steady Shear rate sweep of low concentration PA nanofibers. The Control PA at 2mM was effectively crosslinked with Ca^{2+} at 2 mM PA observed as a substantial increase in the viscosity following ionic crosslinking. The GSH PA at 2mM did not observe a significant difference in viscosity following ionic crosslinking with Ca^{2+}, highlighting the inability to monitor for bulk material changes at low concentration.

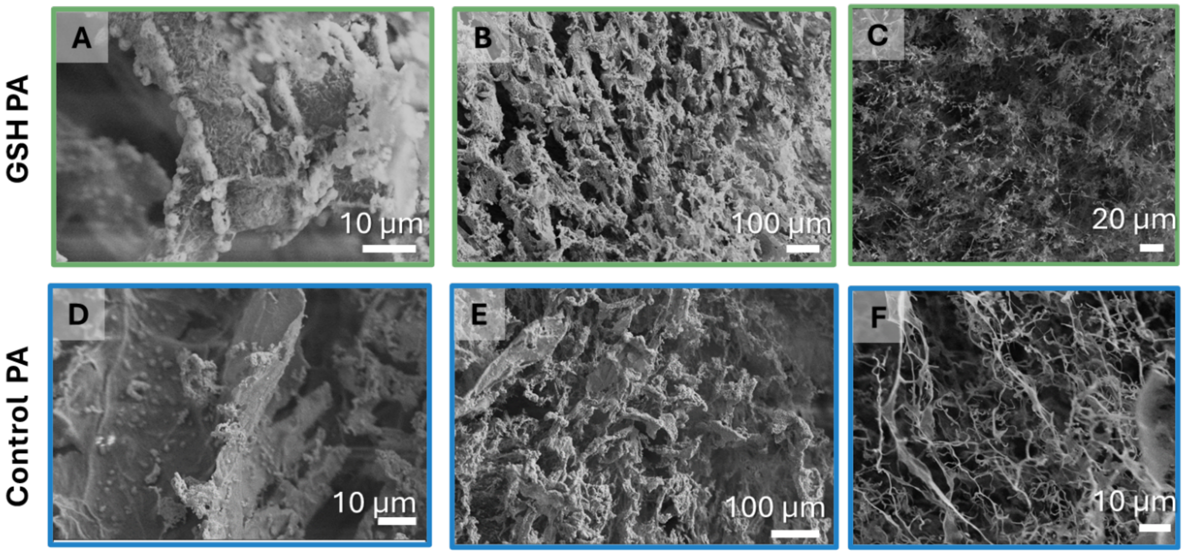

Figure S7. Scanning electron microscopy (SEM) characterization with ionic crosslinking. GSH PA crosslinked with Ca^{2+} demonstrating A) fibers banding together, B) hydrogel scaffold porosity, and C) uncrosslinked GSH PA. Control PA crosslinked with Ca^{2+} demonstrating D) fibers banding together, E) hydrogel scaffold porosity, and F) uncrosslinked Control PA.

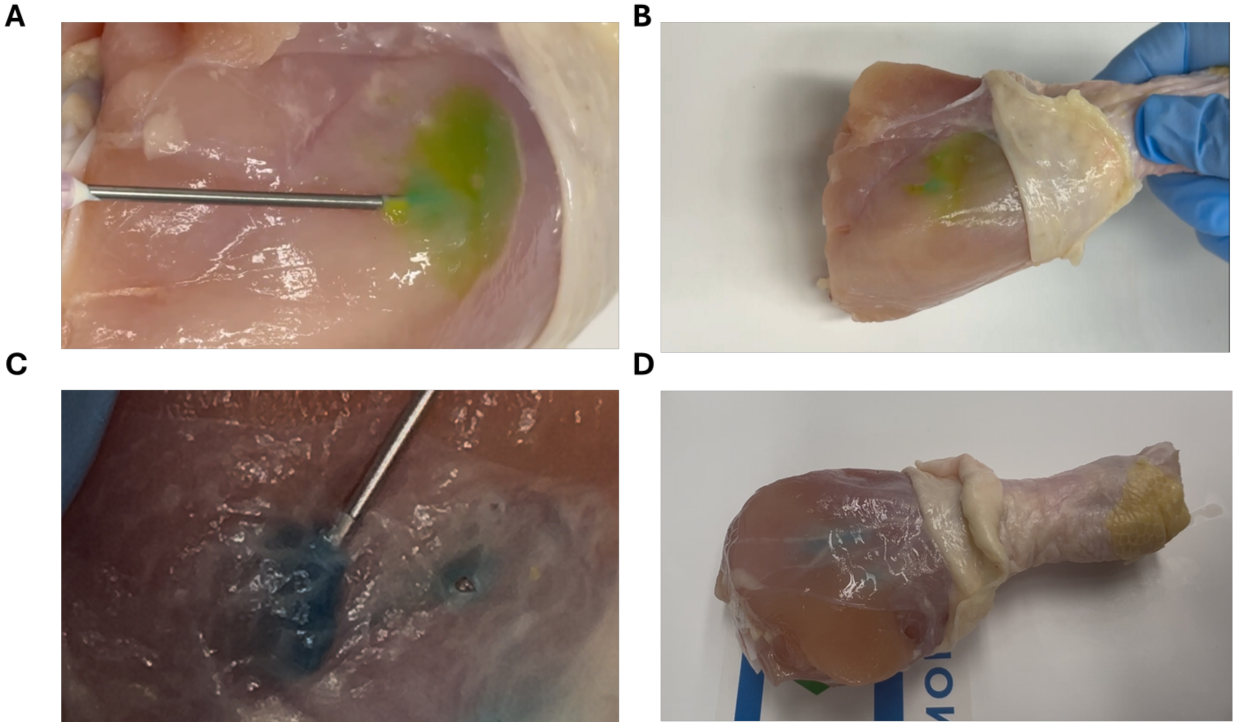

Figure S8. Validation of injectability of the GSH and Control PA into a chicken drumstick. Gelled GSH PA (green) or Control PA (blue) being injected and remaining at site of injury on a chicken drumstick. Injection of 5mM gelled GSH PA A) close up with 18G needle injecting, and B) at lower magnfication following injection. Injection of 5mM gelled Control PA C) close up with 18G needle injecting, and D) at lower magnfication following injection.

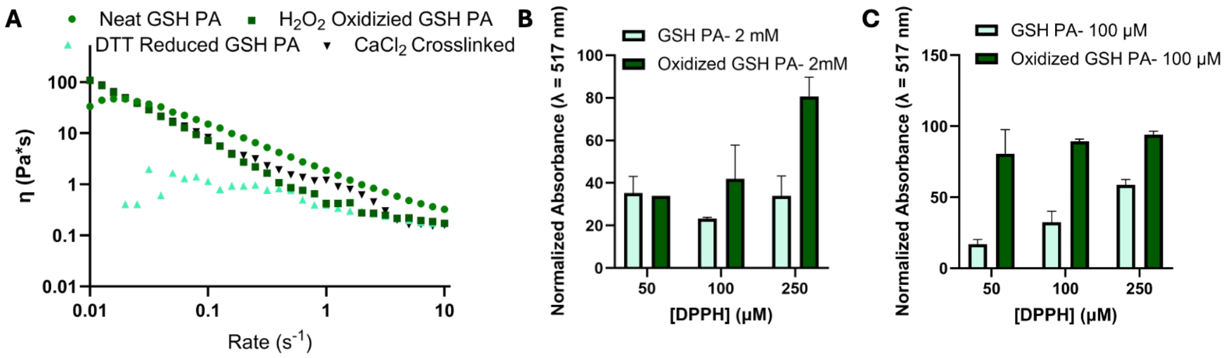

Figure S9. Material properties following oxidation and reduction with H_2O_2/ DTT. A) Steady rate viscosity sweep of 2 mM GSH PA neat, $CaCl_2$ Crosslinked, Oxidized with 2% H_2O_2 for 15 minutes, or reduced with 5 mM DTT for 15 minutes. B) DPPH assay with samples (2mM) from the rheometer, tested with increasing DPPH concentrations, C) DPPH assay with samples from the rheometer, diluted to 100 μM tested with increasing DPPH concentrations.

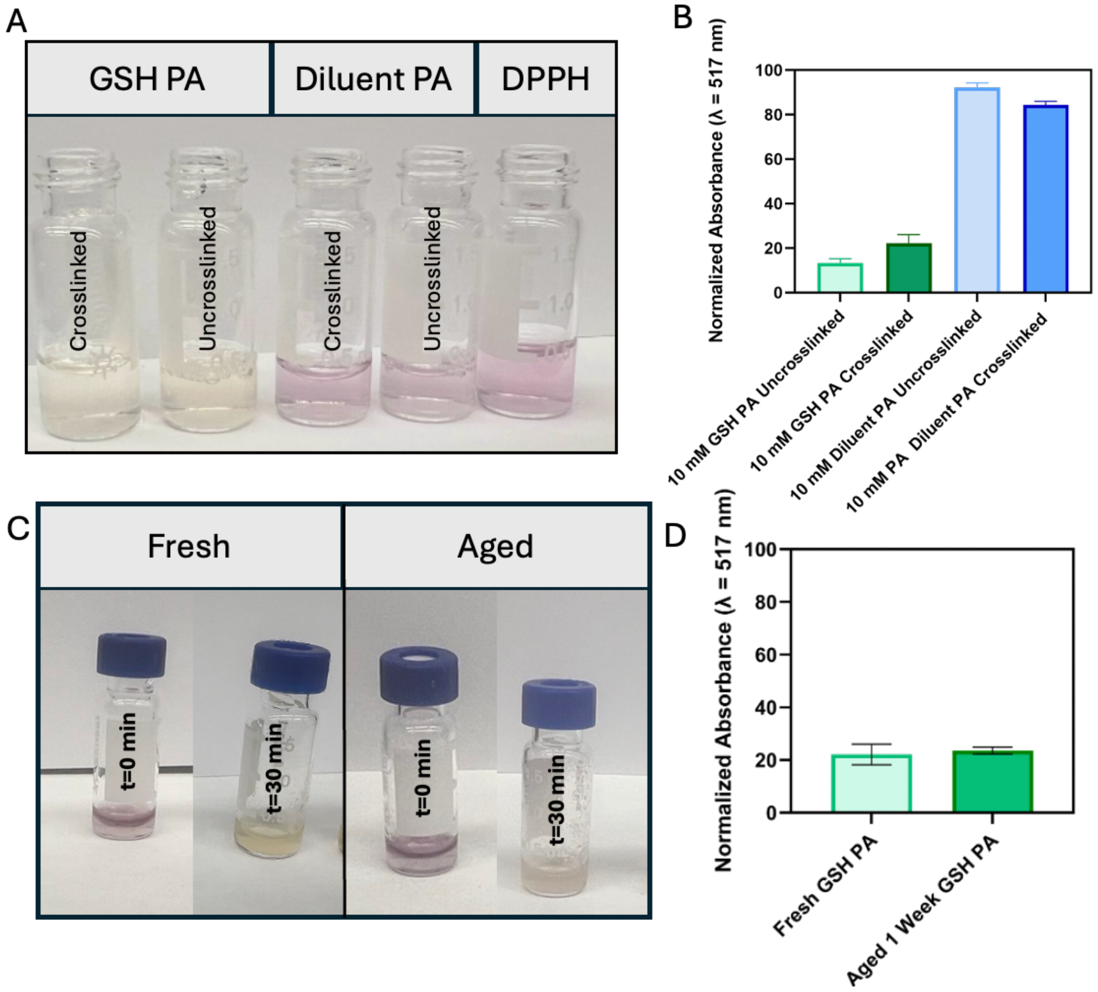

Figure S10. DPPH studies of gelled PAs. Impact of crosslinking on antioxidant capabilities. A) Photo showing quenched and unquenched DPPH following 30-minute incubation (From left to right Crosslinked GSH PA, Uncrosslinked GSH PA, Crosslinked Control PA, Uncrosslinked Control PA, and DPPH control. Samples were diluted to a final DPPH concentration of 62.5 µM to be able to measure absorbance in a plate reader spectrometer. High concentration uncrosslinked PA samples formed organogels following incubation making dilution necessary to read absorbance. All samples underwent same dilution and were immediately read on the plate reader at the completion of the 30min incubation. B) DPPH assay quantifying quenching of crosslinked and uncrosslinked samples, with no significance in GSH PA's radical quenching capabilities following DPPH incubation by student's t-test ($p=0.6288$). C) Photo showing DPPH control (125 µM), and quenched DPPH in both 1-week aged and fresh GSH PA samples with equivalent DPPH added. D) Impact of aging on GSH PA antioxidant activity characterized by DPPH assay, with no significant difference in radical quenching capabilities calculated by a student's t-test (two tailed, assuming equal variance) in GraphPad Prism ($p=0.6678$).

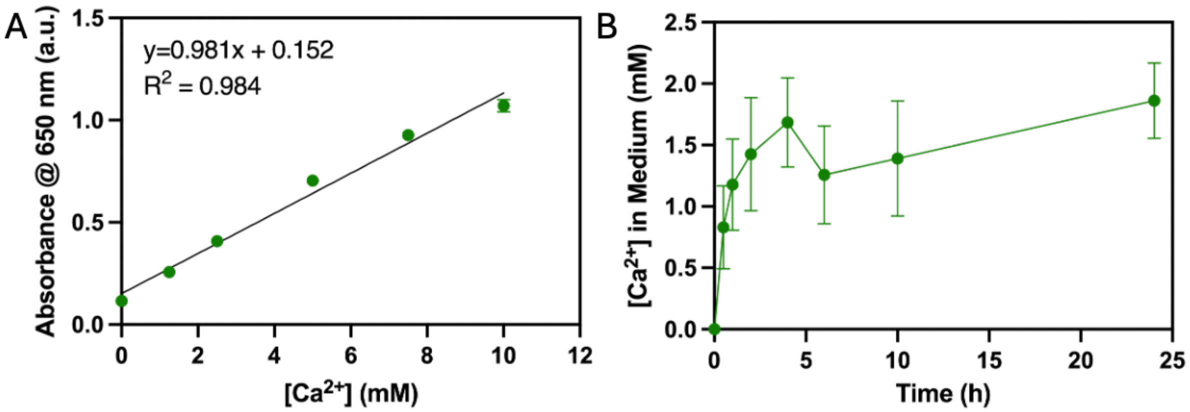

Figure S11. Quantification of Ca^{2+} release from the GSH PA hydrogel. A) Standard curve of the Arsenazo III assay for Ca^{2+} quantification and B) cumulative release of Ca^{2+} from the ionic crosslinked GSH PA hydrogel over a 24 h period at 37°C (total calculated Ca^{2+} concentration within the hydrogel = 5mM).

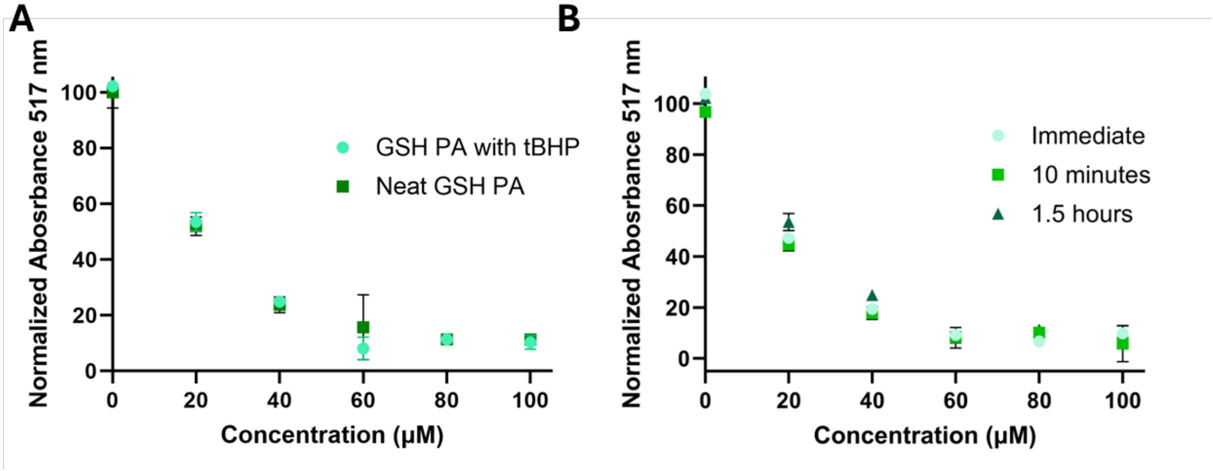

Figure S12. DPPH assay of PAs co-incubated with tBHP. A) Comparing neat GSH to GSH PA that was pre-incubated with 30 µM tBHP for 1.5 hours. No significant difference between treatments was observed highlighting no direct oxidation of the glutathione group occurs from the peroxide-based species. B) DPPH of GSH PA incubated with 30 µM tBHP for various time durations prior to running the DPPH assay. Time points were informed by literature of interactions of tBHP with GSH in rat blood, monitoring times above and below where alterations were observed.[2]

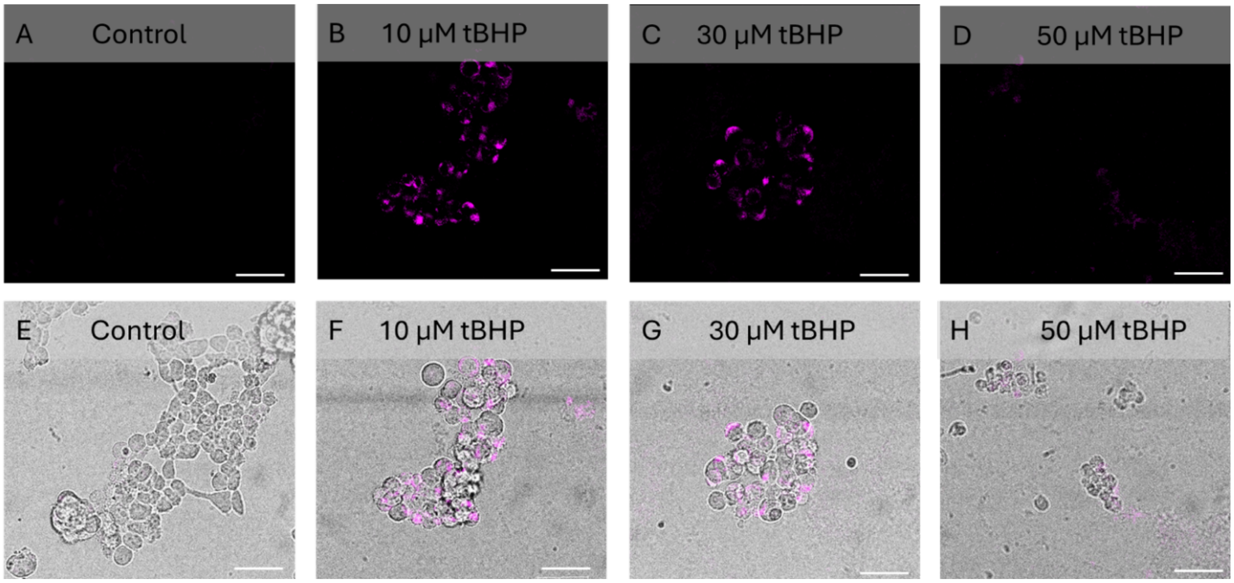

Figure S13. tBHP concentration screening for influence on cellular oxidative stress. CellRox fluorescent imaging, with channel only (top) and brightfield overlay (bottom) for selected tBHP concentrations, highlighting the oxidative damage in cells treated with tBHP at increasing concentrations of tBHP. CellRox only channel for A) no treatment control (i.e. 0 µM tBHP), B) 10 µM tBHP, C) 30 µM tBHP, and D) 50 µM tBHP. Overlay of brightfield and CellRox channels for E) no treatment control (i.e. 0 µM tBHP), F) 10 µM tBHP, G) 30 µM tBHP, and H) 50 µM tBHP.

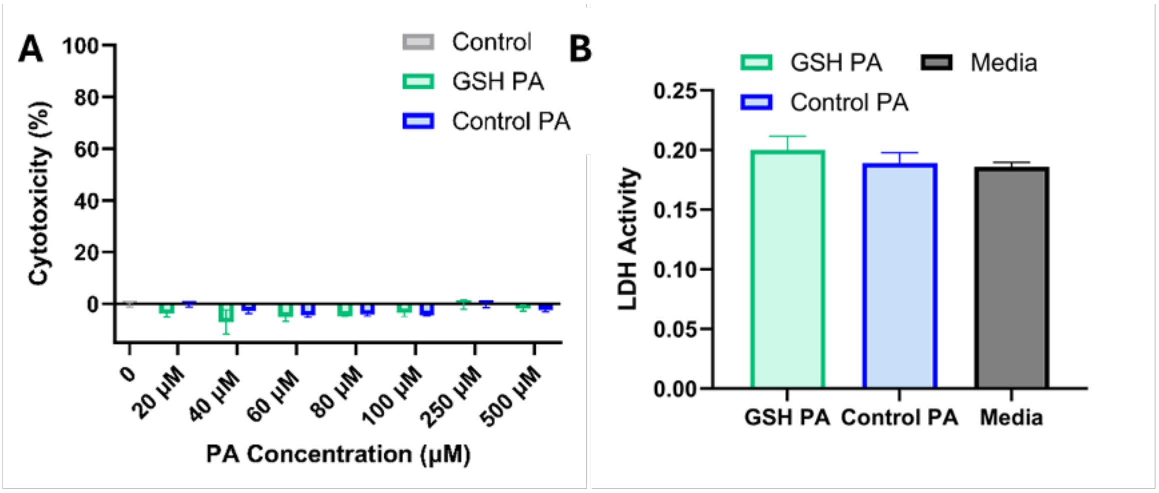

Figure S14. Cytotoxicity of GSH PA and Control PA. A) Cytotoxicity of both the GSH PA and Control PA as assessed by cellular lactate dehydrogenase (LDH) release. B) Comparison of the absorbance of PAs in media at 100 µM- with no cells in the LDH assay confirming that there was no significant interference from the PA nanofibers with LDH and the assay reagents compared to media alone.

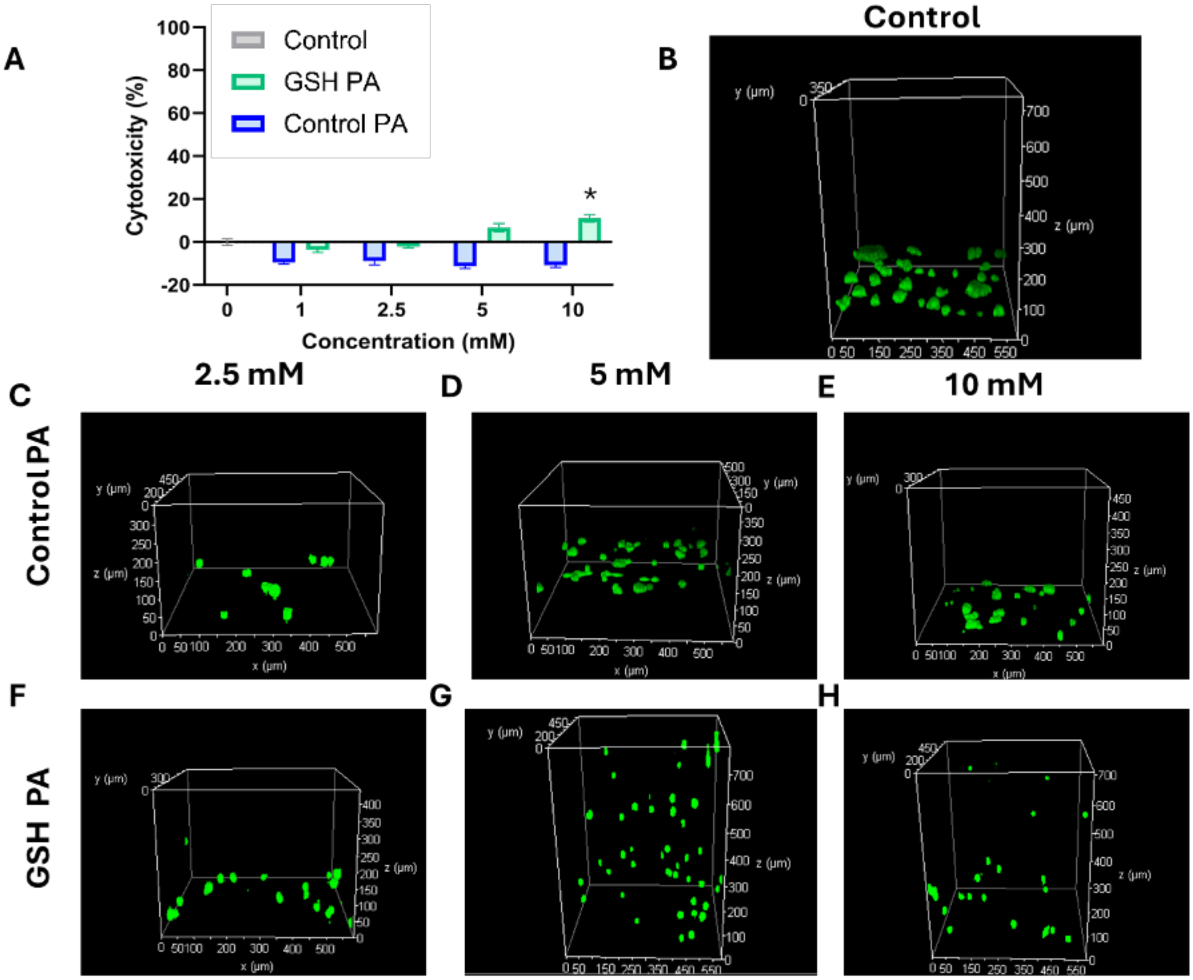

Figure S15. Cytotoxicity of high concentration gelled GSH PA and Control PA. A) Cytotoxicity of both the GSH PA and Control PA as assessed by cellular lactate dehydrogenase (LDH) release from 24-hour coincubation of cells with gelled PAs. * denotes $p < 0.05$ for comparison with control. Significance was determined using a one-way ANOVA, with post hoc Tukey test for means comparison in GraphPad Prism statistical software. Fluorescent confocal microscopy 3-D z-stacks of live cell imaging B) Control C) 2.5 mM Control PA, D) 5 mM Control PA, E) 10 mM Control PA, F) 2.5 mM GSH PA, G) 5 mM GSH PA, H) 10 mM GSH PA.

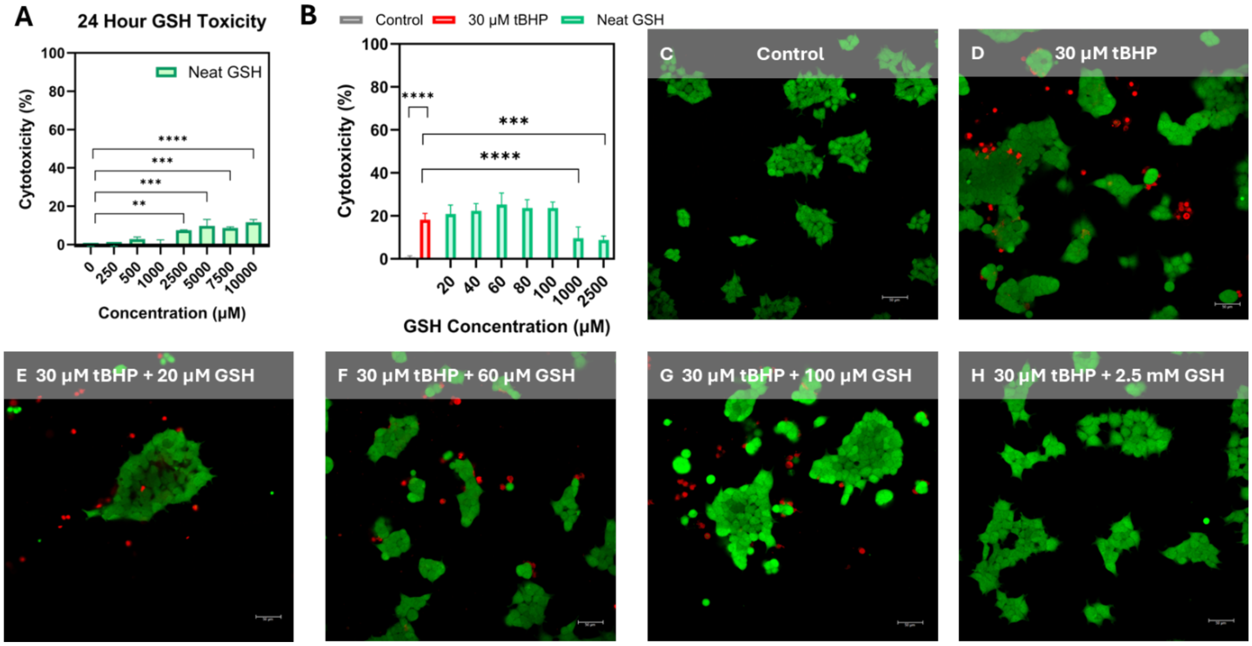

Figure S16. Understanding the impacts of untethered GSH in a tBHP ROS system A) Cytotoxicity of the neat GSH by cellular lactate dehydrogenase (LDH) release from 24-hour coincubation of cells with different concentrations of GSH (n=3, data reported as mean ± s.d.). B) Cytotoxicity of the neat GSH in our ROS assay by cellular lactate dehydrogenase (LDH) release from 24-hour coincubation of cells with different concentration of GSH at 30 µM tBHP (n=6, data reported as mean ± s.d and is the average of two independent triplicates) * denotes $p < 0.05$ for comparison with control. Significance was determined using a one-way ANOVA, with post hoc Tukey test for means comparison in GraphPad Prism statistical software. Live/Dead fluorescent imaging of HEK 293 cells following 24 h of C) Untreated Control, D) 30 µM tBHP, E) 30 µM tBHP + 20 µM GSH, F) 30 µM tBHP + 60 µM GSH, G) 30 µM tBHP + 100 µM GSH, H) 30 µM tBHP + 2500 µM GSH.

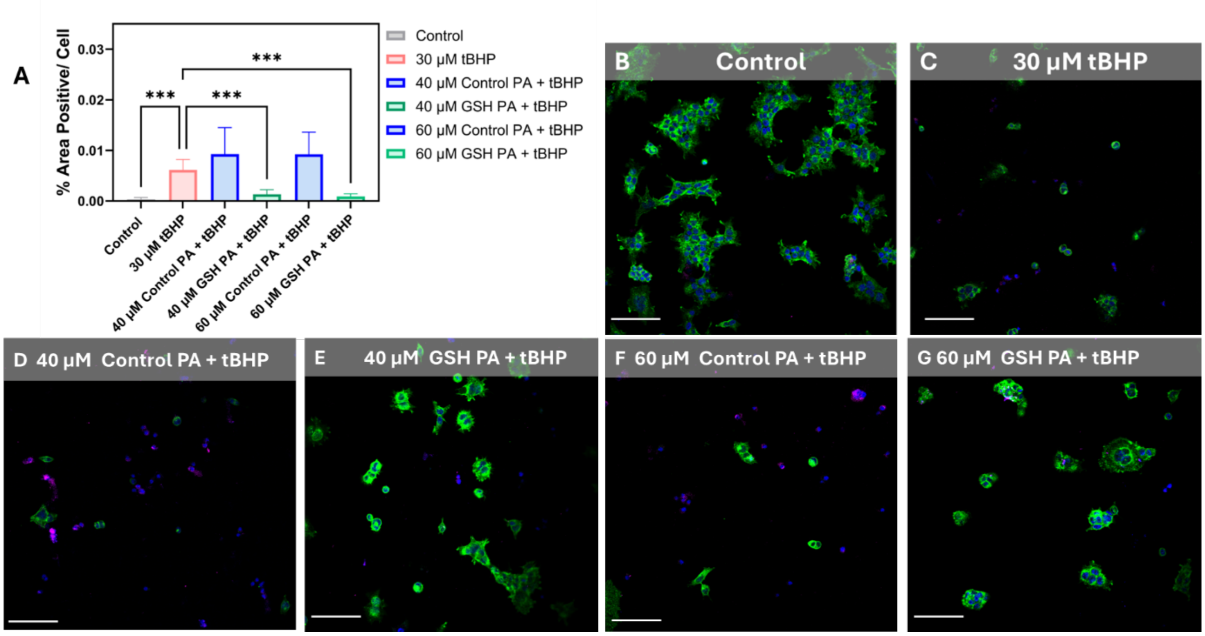

Figure S17. Quantification and representative images used for oxidative stress assessment. A) Secondary quantification of oxidative stress was performed to compare the percentage area of each image that was CellRox positive, normalized by the number of cells per image. This data supports conclusions made from total number of pixels positive per cell. Representative images show number of and general health of cells for B) no treatment control cells, C) 30 µM tBHP, D) 30 µM tBHP + 40 µM Control PA, E) 30 µM tBHP + 40 µM GSH PA, F) 30 µM tBHP + 60 µM Control PA, G) 30 µM tBHP + 60 µM GSH PA. Fluorescent confocal micrographs labelled as nuclei (DAPI, blue), actin (Phalloidin iFluor Actin Stain, green) and oxidative stress (CellRox, magenta).

Supporting Video Captions

Video S1. Phantom injection of the Control PA hydrogel into a chicken drumstick. Demonstration of injection of PA hydrogel through an 18G needle. 1 mL of Control PA was prepared at 5mM concentration with approximately 0.6% by volume blue food coloring added for visualization.

Video S2. Phantom injection of the GSH PA hydrogel into a chicken drumstick. Demonstration of injection of PA hydrogel through an 18G needle. 1 mL of GSH PA was prepared at 5mM concentration with approximately 0.6% by volume green food coloring added for visualization.

Supporting Information References

(1) Chin, S. M.; Synatschke, C. V.; Liu, S.; Nap, R. J.; Sather, N. A.; Wang, Q.; Álvarez, Z.; Edelbrock, A. N.; Fyrner, T.; Palmer, L. C.; et al. Covalent-supramolecular hybrid polymers as muscle-inspired anisotropic actuators. **2018**, *9* (1), 2395.
(2) Simplicio, P. D.; Rossi, R. The time-course of mixed disulfide formation between GSH and proteins in rat blood after oxidative stress with tert-butyl hydroperoxide. **1994**, *1199* (3), 245-252.
